# Genetic Analysis of the *LOXHD1* Gene in Chinese Patients With Non-Syndromic Hearing Loss

**DOI:** 10.3389/fgene.2022.825082

**Published:** 2022-05-27

**Authors:** Wei-Qian Wang, Xue Gao, Sha-Sha Huang, Dong-Yang Kang, Jin-Cao Xu, Kun Yang, Ming-Yu Han, Xin Zhang, Su-Yan Yang, Yong-Yi Yuan, Pu Dai

**Affiliations:** ^1^ Beijing Key Lab of Hearing Impairment Prevention and Treatment, ChinaNational Clinical Research Center for Otolaryngologic DiseasesState Key Lab of Hearing Science, Chinese PLA General Hospital, Chinese PLA Medical School, Ministry of Education, College of Otolaryngology Head and Neck Surgery, Beijing, China; ^2^ Department of Otolaryngology, PLA Rocket Force Characteristic Medical Center, Beijing, China

**Keywords:** *LOXHD1*, DFNB77, non-syndromic hearing loss, next generation sequencing, down-sloping hearing loss

## Abstract

Non-syndromic hearing loss (NSHL) is a common neurosensory disease with an extreme genetic heterogeneity which has been linked to variants in over 120 genes. The *LOXHD1* gene (DFNB77), encoding lipoxygenase homology domain 1, is a rare hearing loss gene found in several populations. To evaluate the importance of *LOXHD1* variants in Chinese patients with NSHL, we performed genetic analysis on *LOXHD1* in 2,901 sporadic Chinese patients to identify the aspect and frequency of *LOXHD1* causative variants. Next-generation sequencing using a custom gene panel of HL was conducted on 2,641 unrelated patients and whole-exome sequencing on the remaining 260 patients. A total of 33 likely causative variants were identified in 21 patients, including 20 novel variants and 13 previously reported pathogenic variants. Each of the 20 novel variants was evaluated according to ACMG criteria. These findings showed that causative variants in *LOXHD1* were found in about 0.72% (21/2,901) of Chinese NSHL patients. This study is by far the largest number of novel variants identified in this gene expanding the range of pathogenic variants in *LOXHD1*, and suggests that variants in this gene occur relatively commonly in Chinese NSHL patients. This extensive investigation of *LOXHD1* in Chinese NSHL patients proposed six recurrent *LOXHD1* variants. These findings may assist in both molecular diagnosis and genetic counseling.

## Introduction

Hearing loss (HL) is common and is known to affect approximately one to three out of 1,000 newborns (Kennedy and Mccann, 2004; Fortnum et al., 2001). HL shows a high degree of clinical and genetic heterogeneity, given different modes of inheritance and the phenotype variabilities associated to the severity, age of onset ranging from congenital and early-onset to late-onset, and large numbers of deafness causing genes. Genetics accounts for over half of the congenital cases. According to the Hereditary Hearing loss Homepage (http://hereditaryhearingloss.org/, accessed 08/2021), there is a total of 124 non-syndromic hearing loss genes that has been identified. Autosomal recessive non-syndromic hearing loss (ARNSHL) accounts for ∼80% of cases of inherited HL. The two most common genes linked to ARNSHL are *GJB2* and *SLC26A4,* observed in multiple populations. In 2009, biallelic variants in *LOXHD1* (lipoxygenase homology domain 1, MIM #613072) was identified as the cause of ARNSHL, DFNB77 (MIM #613079) (Grillet et al., 2009).

Individuals with pathogenic variants in *LOXHD1* usually present with heterogeneous hearing phenotypes. Although *LOXHD1* pathogenic variants tend to be linked to late-onset HL with an onset age of older than 10 years old (yo) (Maekawa et al., 2019), onset may occur at any age from newborn to adult, with considerable variations in severity varying from mild to profound and progression varying from stable to progressive. Recently, the genotype-phenotype correlations of *LOXHD1*-associated HL have been described, indicating that *LOXHD1* variants tend to be associated with a down-sloping audiogram (Maekawa et al., 2019; Kim et al., 2021; Yu et al., 2021).

Ethnicity appears to play a role in determining the *LOXHD1* genetic load in NSHL. In relatively larger cohorts with more than 100 patients, the prevalence of *LOXHD1-*associated HL in United States HL patients was found to be 0.71% (8/1,119) (Sloan-Heggen et al., 2016), 1.5% in the Netherlands (3/200) (Zazo et al., 2017), and 0.97% in Italy (1/103) (Morgan et al., 2018). In 2015, Mori *et al.* reported that patients with pathogenic variants in *LOXHD1* are extremely rare in Japanese HL patients (0.15%, 2/1,314) while in 2019, Maekawa *et al.* identified 28 affected individuals with *LOXHD1* variants among 8,074 Japanese HL patients (0.35%) (Maekawa et al., 2019). A report from Korea indicated that variants in *LOXHD1* accounted for 12.8% of down-sloping sensorineural HL cases (Kim et al., 2021). In 2021, Kim *et al.* reported that in Korean teenagers and young adults with down-sloping audiogram, the prevalence was 33.3% (6/18), considering only late-onset, down-sloping HL (Kim et al., 2021).

According to HGMD Professional database (release 2021.02), 107 pathogenic variants of *LOXHD1* have been identified to be related with HL. To date, over one hundred pathogenic variants in *LOXHD1* have been identified. Although a few sporadic cases of *LOXHD1-*associated HL have been described in Chinese patients (Hu et al., 2018; Shen et al., 2019; Zhang et al., 2019; Bai et al., 2020; Yu et al., 2021; Jin et al., 2022), little is known of the association between the contribution of *LOXHD1* and NSHL in China. Here, our objective was to examine the role of *LOXHD1* in Chinese NSHL patients and to provide a genotypical spectrum in this group of patients.

## Materials and Methods

### Clinical Data

The DNA of 2,901 sporadic NSHL patients belonging to the Han Chinese population was analyzed. The participants were recruited from the Departments of Otolaryngology Head and Neck Surgery, PLA General Hospital, between June 2015 and August 2021, and ranged in age from 6 months to 28 years. All participants presented with bilateral, congenital or late-onset, mild to profound NSHL (age of onset between 0 and 10 years) with no other obvious systemic disorders. Other explanations for HL, such as infection, malformation, or tumors, were ruled out.

The clinical data acquired at the time of assessment included medical history (by questionnaire), temporal bone computed tomography (CT), otoscopy, pure-tone audiometric examination (if over the age of 6 years), auditory steady-state response (ASSR, if below the age of 6 years), acoustic immittance, auditory brainstem responses, and distortion product otoacoustic emission.

HL was assessed using pure-tone hearing thresholds or ASSR response thresholds (0.5, 1, 2, and 4 kHz for both ears). Hearing was scored using the American Speech-Language-Hearing Association (ASHA) guidelines as normal (threshold ≤25 dB), mild HL (threshold: 25.1–40 dB), moderate HL (threshold: 40.1–55 dB), moderately severe HL (threshold: 55.1–70 dB), severe HL (threshold: 70.1–90 dB), and profound HL (threshold >90 dB, including total deafness). The progression of HL was indicated by a 15 dB increase in the hearing or response threshold in one or both ears from one audiogram to the next increased hearing or response threshold. The hearing frequencies were categorized as low (125–500 Hz), mid (1–2 kHz), or high (4–8 kHz). Prelingual HL was defined as that occurring before the development of speech (usually under the age of 3 years), while postlingual loss occurred after speech development (Adam et al., 1993). Evaluations of vestibular function were conducted using the Romberg, caloric, and tandem gait tests.

The audiograms were described according to shape, specifically, down-sloping (≥15 dB difference between the [better] 250–500 Hz thresholds and the 4,000–8,000 Hz thresholds), flat (≤15 dB difference in thresholds from 125 to 8,000 Hz), U-shaped (≥10 dB difference between the poorest mid-frequency [1–2 kHz] threshold and thresholds at higher and lower frequencies), ascending (≥15 dB difference between the 250–500 Hz and the [better] 4–8 kHz thresholds), residual (limited hearing at only a few frequencies), complete deafness (no hearing at maximum outputs regardless of frequency), and unclassified (none of the above).

### Next Generation Sequencing and Bioinformatic Analysis

Genomic DNA was extracted from peripheral blood using a blood DNA extraction kit (TianGen, Beijing, China), as per instructions. Whole-exome capture was applied in 260 patients. A custom capture panel (MyGenostics, Beijing, China) encompassing 3,156 regions of approximately 850 kb target size was constructed to cover the exons and flanking intronic sequences (∼50 bp) for 159 deafness genes (Supplementary Table S1) was applied in 2,641 patients. Using these methods, approximately 20,000 genes were analyzed with WES and 159 genes were analyzed with targeted panel. Detailed descriptions of the gene capture, sequencing, and bioinformatics analyses are given in a previous publication (Yao et al., 2017). CNV Analysis along with both WES and targeted panel was conducted by use of Cnvkit software and was described in detail in a previous publication (Li et al., 2021). For sporadic HL cases, assuming either autosomal dominant (*de novo*) or autosomal recessive inheritance patterns, only variants *de novo* in study participants or variants homozygous or compound heterozygous in study participants and meanwhile heterozygous in their parents were picked as candidate. The variants were comprehensive interpreted using VarSome (Kopanos et al., 2019). Manual variant classification was performed using the American College of Medical Genetics and Genomics (ACMG)/Association for Molecular Pathology (AMP) guidelines for genetic hearing loss (Li et al., 2017). The final screened potential pathogenic variants were verified by Sanger sequencing and validated by parental testing on condition that the DNA samples of parents were available. Some cases in this study have been previously genetically screened as part of the newborn screening of the selected high-risk variants, however, this information is incomplete and was not routinely gathered in this study. Novel variants were defined based on absence from the HGMD professional database, ClinVar database and published literatures.

The data, including phenotypes and observed variants, have been submitted to ClinVar (https://www.ncbi.nlm.nih.gov/clinvar/) under the accession numbers SCV002011797-SCV002011798.

## Results

### Variant Analysis

**FIGURE 1:**
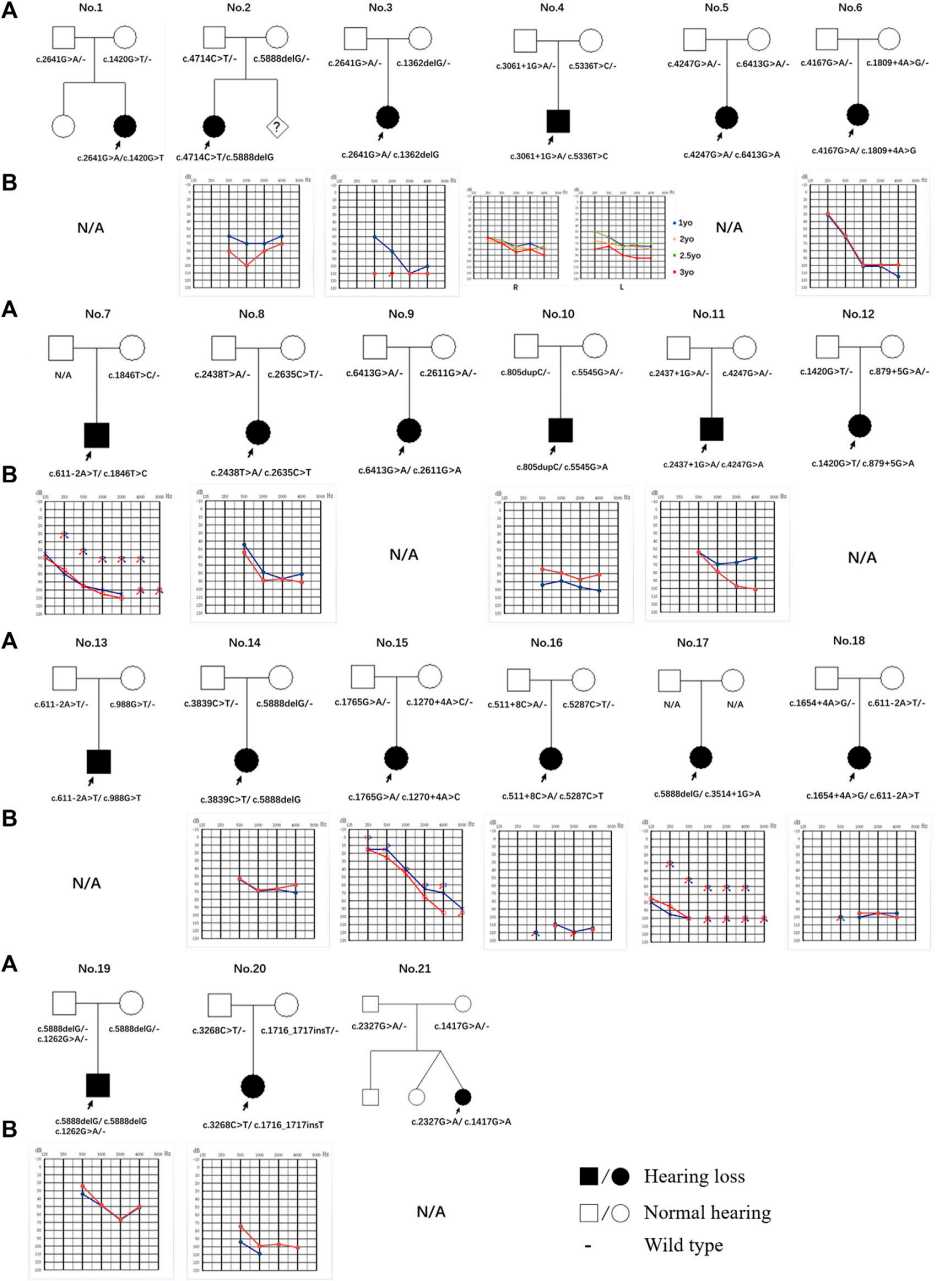
Pedigree and audiogram of families with *LOXHD1* causatives. **(A)** Affected subjects are denoted in black. The proband is indicated by an arrow. **(B)** Audiograms of the affected subjects. (red, right ear; blue, left ear).

In this study, novel variants were defined based on absence from the HGMD professional database, ClinVar database and published literatures. In this case, twenty-one probands (21/2,901; 0.72%) carrying thirty-three likely causative *LOXHD1* variants were identified, including 13 reported pathogenic variants and 20 novel variants. The novel variants included 11 missense variants, 3 nonsense variants, 2 frameshift insertion variants, 1 frameshift deletion variant, and 3 splicing variants. Sixteen novel variants clustered within the PLAT domains, while the remaining four were dispersed in intervals between the PLATs (Figure 1; Table 1). According to ACMG/AMP guidelines, 6 out of 20 novel variants were classified as pathogenic, 1 was classified as likely pathogenic, and 13 were classified as of uncertain significance (Table 1). All the variants identified in the probands were validated in their parents by Sanger sequencing, except patient No.7 (father’s sample was not available) and patient No.17 (parents’ samples were not available). It needs to be mentioned that we detected an average of 350 CNVs per sample, but none met the genetic model of these pedigrees and no *LOXHD1*-associated CNVs were detected.

**TABLE 1:** All possibly causative variants of *LOXHD1* identified in this study (NM_144612.6).

Location	Variant	AA Change	Exon/Intron	Domain	Pathogenicity	ClinVar Included (Yes/No)	Origin	References
chr18:44219571	c.511+8C > A	—	Intron4/39	Interval (PLAT1-2)	Uncertain Significance (PM2+BP4+BP6)	Yes	Chinese	Present study
chr18:44190889	c.611–2A > T	—	Intron5/39	PLAT2	Pathogenic (PVS1+PM1+PM2+PP3)	Yes	Chinese Chinese	Present study Bai, et al. (2020)
chr18:44184147	c.805dupC	p.Leu269ProfsTer2	Exon7/40	PLAT2	Pathogenic (PVS1+PM2+PP3)	No	Chinese	Present study
chr18:44181326	c.988G > T	p.Gly330Trp	Exon8/40	PLAT3	Uncertain Significance (PM2+PP3+BP1)	No	Chinese	Present study
chr18:44174302	c.1262G > A	p.Arg421Gln	Exon9/40	Interval (PLAT3-4)	Uncertain Significance (PM2+BP1+BP4)	No	Chinese	Present study
chr18:44174290	c.1270+4A > C	—	Intron9/39	Interval (PLAT3-4)	Uncertain Significance (PM1+PM2+BP4)	No	Chinese Japanese	Present study Maekawa, et al.,2019
chr18:44173632	c.1362delG	p.Arg455GlyfsTer7	Exon10/40	PLAT4	Pathogenic (PVS1+PM2+PP3)	No	Chinese	Present study
chr18:44173574	c.1420G > T	p.Glu474Ter	Exon10/40	PLAT4	Pathogenic (PVS1+PM2+PP3)	No	Chinese	Present study
chr18:44171892	c.1654+4A > G	—	Intron12/39	Interval (PLAT4-5)	Uncertain Significance (PM2)	No	Chinese	Present study
chr18:44159589	c.1809+4A > G	—	Intron13/39	PLAT5	Uncertain Significance (PM2+BP4)	No	Chinese	Present study
chr18:44146330	c.2327G > A	p.Arg776His	Exon17/40	PLAT6	Uncertain Significance (PM2+BP1+BP4)	Yes	Chinese	Present study
chr18:44146219	c.2437+1G > A	—	Intron17/39	Interval (PLAT6-7)	Pathogenic (PVS1+PM2+PP3)	No	Chinese	Present study
chr18:44143188	c.2438T > A	p.Leu813Ter	Exon18/40	Interval (PLAT6-7)	Likely Pathogenic (PVS1+PM2)	No	Chinese	Present study
chr18:44140496	c.2611G > A	p.Asp871Asn	Exon19/40	PLAT7	Uncertain Significance (PM2+BP1)	No	Chinese	Present study
chr18:44140472	c.2635C > T	p.Arg879Trp	Exon19/40	PLAT7	Uncertain Significance (PM2+PP3+BP1)	No	Chinese	Present study
chr18:44140466	c.2641G > A	p.Gly881Arg	Exon19/40	PLAT7	Likely Pathogenic (PS1+PM2)	No	Chinese Dutch	Present study Wesdorp M, et al.,2018
chr18:44140045	c.3061+1G > A	—	Intron19/39	PLAT7	Pathogenic (PVS1+PM1+PP5+PM2+PP3)	No	Chinese Dutch	Present study Zazo, et al. (2017)
chr18-44137401	c.3268C > T	p.Arg1090Trp	Exon21/40	PLAT7	Uncertain Significance (PM1+PM2+PP3+BP1)	No	Chinese Chinese	Present study Liu et al. (2020)
chr18:44126857	c.3514+1G > A	—	Intron22/39	Interval (PLAT7-8)	Pathogenic (PVS1+PM2+PP3+PP5)	Yes	Chinese	Present study
chr18:44121813	c.3839C > T	p.Ala1280Val	Exon25/40	PLAT8	Uncertain Significance (PM2+BP1+BP4)	No	Chinese	Present study
chr18:44114343	c.4167G > A	p.Trp1389Ter	Exon27/40	PLAT9	Pathogenic (PVS1+PM2+PP3)	No	Chinese	Present study
chr18:44114293	c.879+5G > A (NM_001145472.2) [c.4212+5G > A (NM_144612.6)]	—	Intron9/23 [Intron27/39]	PLAT9	Uncertain Significance (PM1+PM2+PP5)	Yes	Chinese Chinese	Present study Yu, et al. (2021)
chr18:44113253	c.4247G > A	p.Trp1416Ter	Exon28/40	PLAT9	Pathogenic (PVS1+PM1+PM2+PP3+PP5)	Yes	Chinese Chinese	Present study Yu, et al. (2021)
chr18:44104697	c.4714C > T	p.Arg1572Ter	Exon30/40	PLAT10	Pathogenic (PVS1+PS1+PP5+PM2+PP3)	Yes	Chinese Jewish	Present study Edvardson S, et al.,2011
chr18:44102100	c.1716_1717insT (NM_001145472.2) [c.5049_5050insT (NM_144612.6)]	p.Ala573CysfsTer16 [p.Ala1684CysfsTer16]	Exon14/24 [Exon32/40]	PLAT11	Pathogenic (PVS1+PM2+PP3)	No	Chinese	Present study
chr18:44101233	c.1765G > A (NM_001145472.2) [c.5085 + 831G > A (NM_144612.6)]	p.Gly589Arg	Exon15/24 [Intron32/39]	PLAT11	Uncertain Significance (PM2+PP3+BP1)	No	Chinese	Present study
chr18:44101152	c.1846T > C (NM_001145472.2) [c.5085 + 912T > C (NM_144612.6)]	p.Cys616Arg	Exon15/24 [Intron32/39]	PLAT11	Uncertain Significance (PM2+PP3+BP1)	No	Chinese	Present study
chr18:44089705	c.5287C > T	p.Arg1763Trp	Exon34/40	PLAT12	Uncertain Significance (PM2+PP3+BP1)	No	Chinese	Present study
chr18:44087671	c.5336T > C	p.Leu1779Pro	Exon35/40	PLAT12	Uncertain Significance (PM2+BP1+BP4)	No	Chinese	Present study
chr18:44085948	c.5545G > A	p.Gly1849Arg	Exon36/40	PLAT13	Uncertain Significance (PM1+PM2+PP3+BP1)	No	Chinese Czech	Present study Plevova P, et al.,2017
chr18:44065090	c.5888delG	p.Gly1963AlafsTer136	Exon38/40	PLAT14	Pathogenic (PVS1+PS1+PM2+PP3+PP5)	No	Chinese Chinese	Present study Bai, et al. (2020)
chr18:44057658	c.6413G > A	p.Arg2138Gln	Exon40/40	PLAT15	Uncertain Significance (PM2+PP3+BP1)	No	Chinese	Present study
chr18:44057557	c.1417G > A (NM_001145473.2) [c.6514G > A (NM_144612.6)]	p.Val473Met [p.Val2172Met]	Exon9/9 [Exon40/40]	PLAT15	Uncertain Significance (PM2+PP3+BP1)	No	Chinese	Present study

The candidate variants selected from the sequencing data are shown in Supplementary Table S2. The variants that co-segregated in study participants and parents are shown in bold, and were classified as potentially causative. In patient No.19, a reported variant c.5888delG (p.Gly1963AlafsTer136) was detected in the homozygous state in the patient together with a novel heterozygous variant c.1262G > A (p.Arg421Gln). The two variants were *in cis* in his father whose hearing was normal. The variant c.5888delG in *LOXHD1* leading to a premature termination codon was coded as “PVS1+PS1+PM2+PP3+PP5” and listed as “pathogenic” as mentioned above, while c.1262G > A leading to a single amino acid substitution was coded as “PM2+BP1+BP4” and listed as of “uncertain significance”. Therefore, we presumed c.5888delG was the possible causative variant in patient No.19. Patient No.1 showed two heterozygous variants (c.2641G > A and c.1420G > T) in *LOXHD1*, in addition to two compound heterozygous variants (c.1256A > T and c.1255A > C) in *HARS1*. Given that the two *HARS1* variants were coded as “c.1256A > T: PM2+PP3” and “c.5557C > G: PM2+BP4” and listed as “Uncertain significance” according to the ACMG criteria with the same population frequency of 0.00109 in East Asia, and a lack of reports of their possible pathogenic significance, we assumed *HARS1* was not the causative gene in patient No.1. Similarly, in patient No.8, in addition to the compound heterozygous variants (c.2438T > A and c.2635C > T) in *LOXHD1*, variants (c.3658G > A and c.5557C > G) in *MYO15A* were identified, with the parents as carriers. There are no reported pathogenic associations of these *MYO15A* variants, and the ACMG codes are “c.3658G > A: PP2+BS1+BS2+BP4” and “c.5557C > G: PM1+PM2+PP2+BP4”; thus, they were listed as variants of benign and uncertain significance, respectively. *MYO15A* was, therefore, not considered to be the causative gene in this family. Nevertheless, as it is possible that these variants may have influenced HL, their relevance cannot be discounted altogether and further investigation should be undertaken. In patient No.7, compound heterozygous variants (c.611–2A > T and c.1846T > C) in *LOXHD1* were identified in the affected proband and c.611–2A > T was confirmed as inherited from his mother; the c.1846T > C variant could not be classified as *de novo* or not as the father’s blood sample was not available. A similar situation was seen in patient No.17, where the parents’ blood samples were not available.

### Recurrent Variant

Of all the variants in *LOXHD1* identified in this study, a total of six variants were recurrent, including four reported (c.5888delG, c.611-2A > T, c.2641G > A, and c.4247G > A) and two novel (c.1420G > T and c.6413G > A) variants. Among the reported variants, c.5888delG was detected in three unrelated patients in the heterozygous state (Nos.2, 14, and 17) and in one patient in the homozygous state (No.19), and c.611-2A > T was detected in three unrelated patients in the heterozygous state (Nos.8, 14, and 19). Both c.5888delG and c.611-2A > T were defined as “pathogenic” according to the ACMG criteria. The frequency of c.5888delG in the Chinese NSHL population was estimated as 0.0862% (5/5,802), which is four times higher than the value of 0.000183 for the East Asian population recorded in the gnomAD Exomes database. The frequency of c.611-2A > T in the Chinese NSHL population was estimated as 0.0517% (3/5,802) and it was not found in the gnomAD exomes or gnomAD genomes databases. The other four variants, c.2641G > A, c.4247G > A, c.1420G > T, and c.6413G > A, were detected in two unrelated patients separately in the heterozygous state and their frequencies in the Chinese NSHL population were estimated as 0.0345% (2/5,802). The variants c.2641G > A, c.4247G > A and c.1420G > T were defined as “likely pathogenic”, “pathogenic”, and “pathogenic”, respectively, according to the ACMG criteria and their frequencies in the East Asian population were not found in either the gnomAD exomes database or the gnomAD genomes database. Although c.6413G > A was classified as a variant of “uncertain significance” according to the ACMG criteria, it is noteworthy that the variant frequency is recorded as 0.033 in the East Asian population in the gnomAD exomes database, which indicated that this variant is likely to be benign.

### Clinical Features of Patients and Relationships Between Genotypes and Phenotypes

Table 2 lists the clinical features of the 21 unrelated participants with *LOXHD1* compound heterozygous/homozygous variants, including age of onset, visiting age, audiogram classification, HL progression, and vestibular symptoms. The age of onset varied from newborn to 10 years, with most participants showing evidence of congenital HL between the ages of 0–3 years (19/21, 90.5%). There were no vestibular symptoms present in any of the participants.

**TABLE 2:** Clinical features and *LOXHD1* pathogenic variant combinations of affected probands identified in the present study (NM_144612.6).

Patient No	Variant 1	Variant 2	Phenotype	Age of Visiting	Age of Onset	Progression		Symmetry	Vertigo
1	c.2641G > A (p.Gly881Arg)	c.1420G > T (p.Glu474Ter)	Hearing condition obviously deteriorated at 21 years old. Audiogram is not available	24yo	10yo		Yes	—	No
2	c.5888delG (p.Gly1963AlafsTer136)	c.4714C > T (p.Arg1572Ter)	Flat audiogram configuration with thresholds of 65 dB on the left ear at 4 years old. U-shaped audiogram configuration with a threshold of 100 dB at the 1 kHz frequency on the right ear at 4 years old	4yo	1yo		No	asymmetric	No
3	c.1362delG (p.Arg455GlyfsTer7)	c.2641G > A (p.Gly881Arg)	Down-sloping audiogram configuration on the left ear at 4 years old. Flat audiogram configuration with thresholds of about 110 dB on the right ear at 4 years old	4yo	0yo		---	asymmetric	No
4	c.3061+1G > A	c.5336T > C (p.Leu1779Pro)	Down-sloping audiogram configuration with thresholds of about 70 dB on both ears at 1 year old. The thresholds progressed on the left ear at 3 years old	3yo	0yo		Yes	symmetric	No
5	c.4247G > A (p.Trp1416Ter)	c.6413G > A (p.Arg2138Gln)	Audiogram is not available	2.5yo	1 yo		—	—	No
6	c.4167G > A (p.Trp1389Ter)	c.1809+4A > G	Down-sloping audiogram configuration with thresholds of 30 dB at the 250 Hz frequency and 100 dB at the 1–4 kHz frequencies on both ears	1.5yo	0yo		—	symmetric	No
7	c.611–2A > T	c.1846T > C^a^ (p.Cys616Arg)	Hearing impairment was noticed at 1 year old. Down-sloping audiogram configuration with thresholds of 60 dB at the 125 Hz frequency and over 90 dB at the 500 Hz-8 kHz frequencies on both ears at 28 years old	28yo	1yo		Yes	symmetric	No
8	c.2438T > A (p.Leu813Ter)	c.2635C > T (p.Arg879Trp)	Down-sloping audiogram configuration with thresholds of about 50 dB at the 500 Hz frequency and about 85 dB at the 1–4 kHz frequencies on both ears at 7 years old	7yo	5yo		No	symmetric	No
9	c.6413G > A (p.Arg2138Gln)	c.2611G > A (p.Asp871Asn)	Bilateral profound hearing loss with good feedback after unilateral cochlear implantation. Audiogram is not available	4yo	0yo		—	symmetric	No
10	c.805dupC (p.Leu269ProfsTer2)	c.5545G > A (p.Gly1849Arg)	Down-sloping audiogram configuration with thresholds of about 90 dB on the left ear and about 80 dB on the right ear at 4 years old. Bilateral profound hearing loss with good feedback after unilateral cochlear implantation	4yo	1yo		—	symmetric	No
11	c.4247G > A (p.Trp1416Ter)	c.2437+1G > A	Down-sloping audiogram configuration with thresholds of 55 dB at the 500 Hz frequency and 100 dB at the 4 kHz frequency on the right ear at 3 years old. Slight down-sloping audiogram configuration with thresholds of about 60 dB on the left ear at 3 years old	3yo	2yo		No	asymmetric	No
12	c.1420G > T (p.Glu474Ter)	c.879+5G > A^a^	Bilateral profound hearing loss with satisfactory speech development after unilateral cochlear implantation. Audiogram is not available	4yo	3yo		—	symmetric	No
13	c.988G > T (p.Gly330Trp)	c.611–2A > T	Bilateral profound hearing loss with satisfactory speech development after unilateral cochlear implantation. Audiogram is not available	3yo	2yo		Yes	symmetric	No
14	c.5888delG (p.Gly1963AlafsTer136)	c.3839C > T (p.Ala1280Val)	Down-sloping audiogram configuration with thresholds of about 60 dB on both ears at 6 months old	5yo	0yo		No	symmetric	No
15	c.1765G > A^a^ (p.Gly589Arg)	c.1270+4A > C	Down-sloping audiogram configuration with thresholds of about 20 dB at the 250–500 Hz frequencies and about 90 dB at the 8 kHz frequency on both ears at 5 years old	5yo	2yo		No	symmetric	No
16	c.5287C > T (p.Arg1763Trp)	c.511+8C > A	Flat audiogram configuration with thresholds of over 110 dB on both ears at 1.5 years old	1.5yo	1yo		---	symmetric	No
17	c.5888delG (p.Gly1963AlafsTer136)	c.3514+1G > A	Hearing impairment was noticed at 2 years old. Down-sloping audiogram configuration with thresholds of about 80 dB at the 125 Hz frequency and over 100 dB at the 500 Hz-8 kHz frequencies on both ears at 28 years old	28yo	2yo		—	symmetric	No
18	c.1654+4A > G	c.611–2A > T	Flat audiogram configuration with thresholds of over 90 dB on both ears at 1 year old	1yo	1yo		—	symmetric	No
19	c.5888delG (p.Gly1963AlafsTer136)	c.5888delG (p.Gly1963AlafsTer136)	Down-sloping audiogram configuration with thresholds of about 30 dB at the 500 Hz frequency, 70 dB at the 2 kHz frequency and 50 dB at the 4 kHz frequency on both ears	0.5yo	0yo		—	symmetric	No
	c.1262G > A (p.Arg421Gln)						—		
20	c.1716-1717insT^a^ (p.Ala573CysfsTer16)	c.3268C > T (p.Arg1090Trp)	Down-sloping audiogram configuration with thresholds of 75dB at the 500 Hz frequency and about 100dB at the 1–4 kHz frequencies on the right ear. Similar audiogram configuration with average thresholds 10–20dB lower on the left ear. Satisfactory speech development after unilateral cochlear implantation	2yo	0yo		No	symmetric	No
21	c.1417G > A^b^ (p.Val473Met)	c.2327G > A (p.Arg776His)	Bilateral profound hearing loss with satisfactory speech development after bilateral cochlear implantation. Audiogram is not available	2yo	2yo		No	symmetric	No

a The version of NM_001145472.2.

b the version of NM_001145473.2.

By evaluating of the types of HL among the 15 probands with available audiograms, relationships between genotype and phenotype could be determined (Figure 1). The HL in patient No.4 (male/3 yo), No.6 (female/1.5 yo), No.7 (male/28 yo), No.8 (female/7 yo), No.10 (male/4 yo), No.14 (female/0.5 yo), No.15 (female/5 yo), No.17 (female/28 yo), No.19 (male/0.5 yo), and No.20 (female/2 yo) showed symmetric down-sloping audiogram configurations (10/15, 66.7%). The HL in patient No.16 (female/1.5 yo) and No.18 (female/1 yo) showed flat audiogram configurations with average thresholds over 90 dBHL on both ears at the age of onset (2/15, 13.3%). Patient No.3 (female/4 yo) and No.11 (male/3 yo) showed asymmetric HL with a down-sloping audiogram configuration on one ear and a flat audiogram configuration on the other ear (2/15, 13.3%). Patient No.2 (female/4 yo) had asymmetric HL with a flat audiogram configuration on the left ear and a U-shaped audiogram configuration on the right ear (1/15, 6.7%). Besides these, patients No.9, No.12, No.13, and No.21 who had been diagnosed with profound bilateral NSHL had already received unilateral or bilateral cochlear implantation and the follow-up feedback showed satisfactory speech development after implantation.

Of the 11 patients with follow-up information, four complained of progressive HL while seven gave feedback of stable hearing status. Patient No.4, the only patient with multiple hearing examination results, diagnosed with bilateral symmetric severe hearing loss at 1 yo, showed progressive HL on the left ear and a relatively stable hearing status after 2 years of follow-up (Figure 1; Table 2).

## Discussion

It was observed that pathogenic *LOXHD1* variants were associated with ∼0.72% (21/2,901) of sporadic NSHL cases in a patient cohort without apparent disorders in other systems. All the *LOXHD1* variant–positive patients were under 30 years old and 90.5% (19/21) had HL of congenital onset. In total, 13 reported pathogenic variants and 20 novel variants of *LOXHD1* were identified including six recurrent variants.

The protein LOXHD1 is known to be associated with the auditory system. The mouse *Loxhd1* homologous to human *LOXHD1* was first cloned by Grillet et al., in 2009 and was found to be expressed in the cochlear and vestibular hair cells of young mice and in hair cells along the length of the stereocilia in adult mice (Liu et al., 2020; Grillet et al., 2009; Trouillet et al., 2021). Recently, *Loxhd1* was identified as being required for the mechanotransduction process by which hair cells convert physical forces into electrochemical responses (Trouillet et al., 2021). To date, 19 RefSeq transcripts along with 19 RefSeq proteins have been identified (https://www.ncbi.nlm.nih.gov/gene/?term=LOXHD1). The LOXHD1 protein is highly evolutionarily conserved, and consists of 15 PLAT domains (NM_144612, NP_653213) or 16 PLAT domains (NM_001384474, NP_001371403) and the relevant versions used have been adjusted to transcripts variant 1 (NM_144612) in this study. The assumed function of the PLAT domain is to facilitate access to the plasma membrane in a Ca^2+^-dependent manner (Bateman and Sandford, 1999; Lemmon, 2008). Although the functions of *LOXHD1* have not been fully elucidated, it can be roughly inferred from the function of the PLAT domain as the protein is made up entirely of PLAT repeats. The variants identified in our study were distributed in 14 of the 15 domains and their intervals, with most variants concentrated in the PLAT7 domain.

**FIGURE 2:**
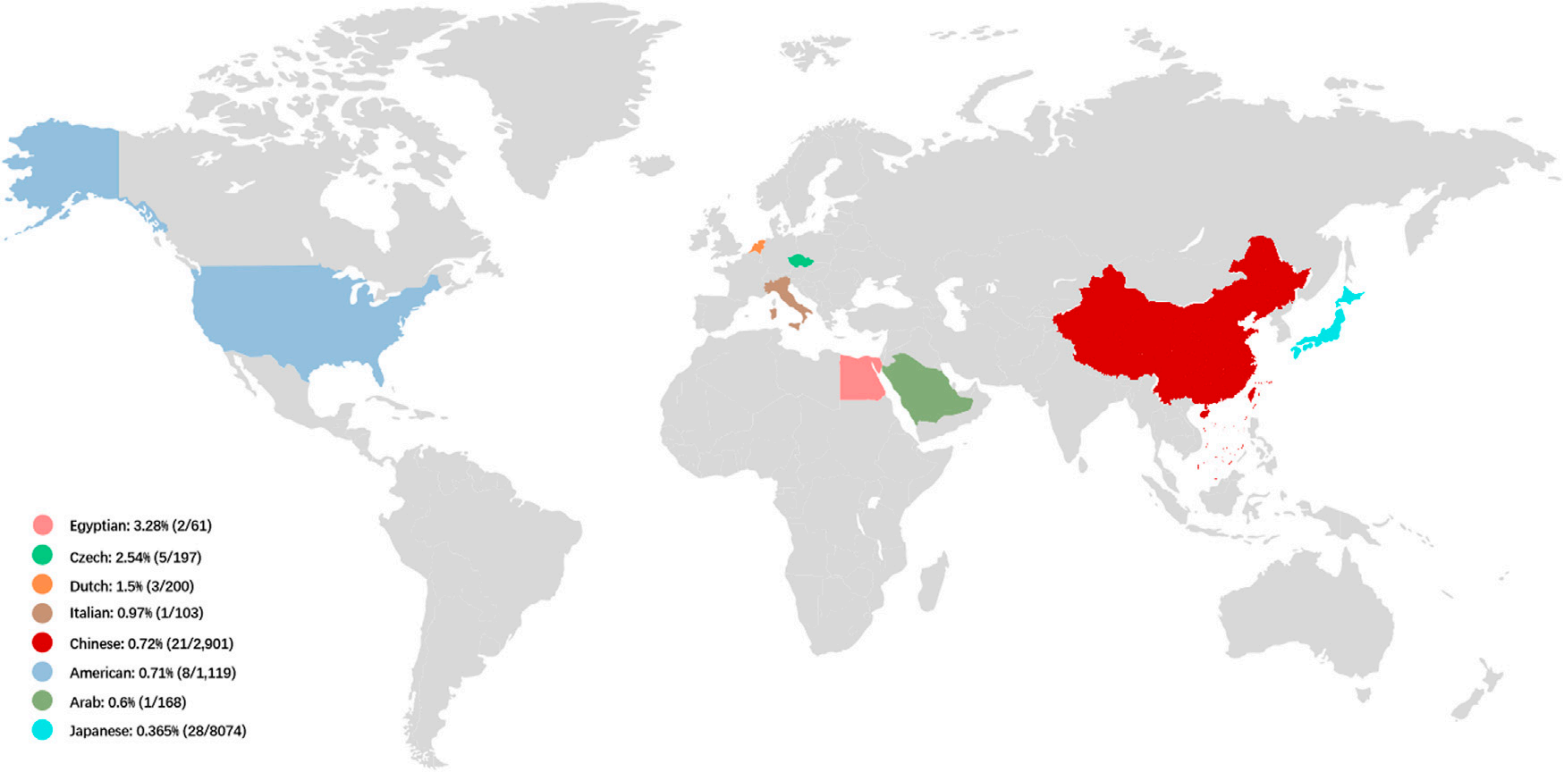
The prevalence of *LOXHD1*-associated HL in NSHL cohorts in different countries. The prevalence of *LOXHD1*-associated HL in NSHL cohorts ranged rom 0.365 to 3.28% in the seven countries (Egypt, Czech, Netherlands, Italy, China, United States, Arab and Japan).

NSHL typified by a down-sloping audiogram configuration suggests *LOXHD1* involvement, and the phenotype and inheritance patterns are similar to those seen with *SLC26A4* and *TMPRSS3*. In our study, most cases showed congenital HL (19/21, 90.5%) and bilateral symmetric down-sloping audiogram configurations (10/15, 66.7%) that were in accordance with previous reports (Kim et al., 2021; Bai et al., 2020). To date, the prevalence of *LOXHD1*-associated HL in relatively larger NSHL cohorts with more than 50 patients has been reported only in seven countries, ranging from 0.365 to 3.28% (Figure 2). Our results also indicate that *LOXHD1* variants account for about 0.72% (21/2,901) of NSHL in Chinese patients, an incidence similar to that reported in the American population (0.71%, 8/1,119) (Bateman and Sandford, 1999; Lemmon, 2008). In order to analyze the correlation between variant location within or out of domain and hearing phenotype, we grouped the 21 patients into “PLAT-domain and PLAT-domain”, “PLAT-domain and interval”, and “interval and interval”. There were 15 patients in the group “PLAT-domain and PLAT-domain” with two variants both locating within the PLAT domains, six patients in the group “PLAT-domain and interval” with one variant locating within a PLAT domain and the other variant within an interval, and no patients in the group “interval and interval”. There seemed to be no difference in the hearing threshold between groups due to large variability between individuals (Supplementary Figure S1-Type1). Of all 33 variants identified in this study, 12 were non-truncating variations (36.4%) and 21 were truncating variations (63.6%). Similarly, we grouped the 21 patients into “truncating and truncating”, “non-truncating and truncating” and “non-truncating and non-truncating” to analyze the relationship between variant severity and hearing phenotype. There were 7 patients in the group “truncating and truncating” carrying two truncating variations, 2 patients in the group “non-truncating and non-truncating” carrying two non-truncating variations, and 12 patients in the group “non-truncating and truncating” carrying both types of variations simultaneously. Again, we noticed no difference between the groups in terms of hearing threshold due to the large variability between individuals (Supplementary Figure S1-Type2). Besides, we counted the number of different types of variants in *LOXHD1* to analyze their proportions of occurrence. Of the 124 variants of *LOXHD1* worldwide, 63 are missense variants, 22 are nonsense variants, 16 are frameshifting variants, 12 are splice-site variants, 9 are intronic variants and 2 are synonymous variants. Of all these variants, 51 were found in the Chinese population, including 27 missense variants, 7 nonsense variants, 6 frameshifting variants, 5 splice-site variants, and 6 intronic variants. The proportions of different types of variation of *LOXHD1* both worldwide and in China were shown in Supplementary Figure S2 and the results suggested that *LOXHD1*-associated hearing loss is caused firstly by missense variants and secondly by nonsense variants, which were consistent with the previous work that the majority of loss of function (LoF) variants in *LOXHD1* are nonsense (Azaiez et al., 2018).

**FIGURE 3:**
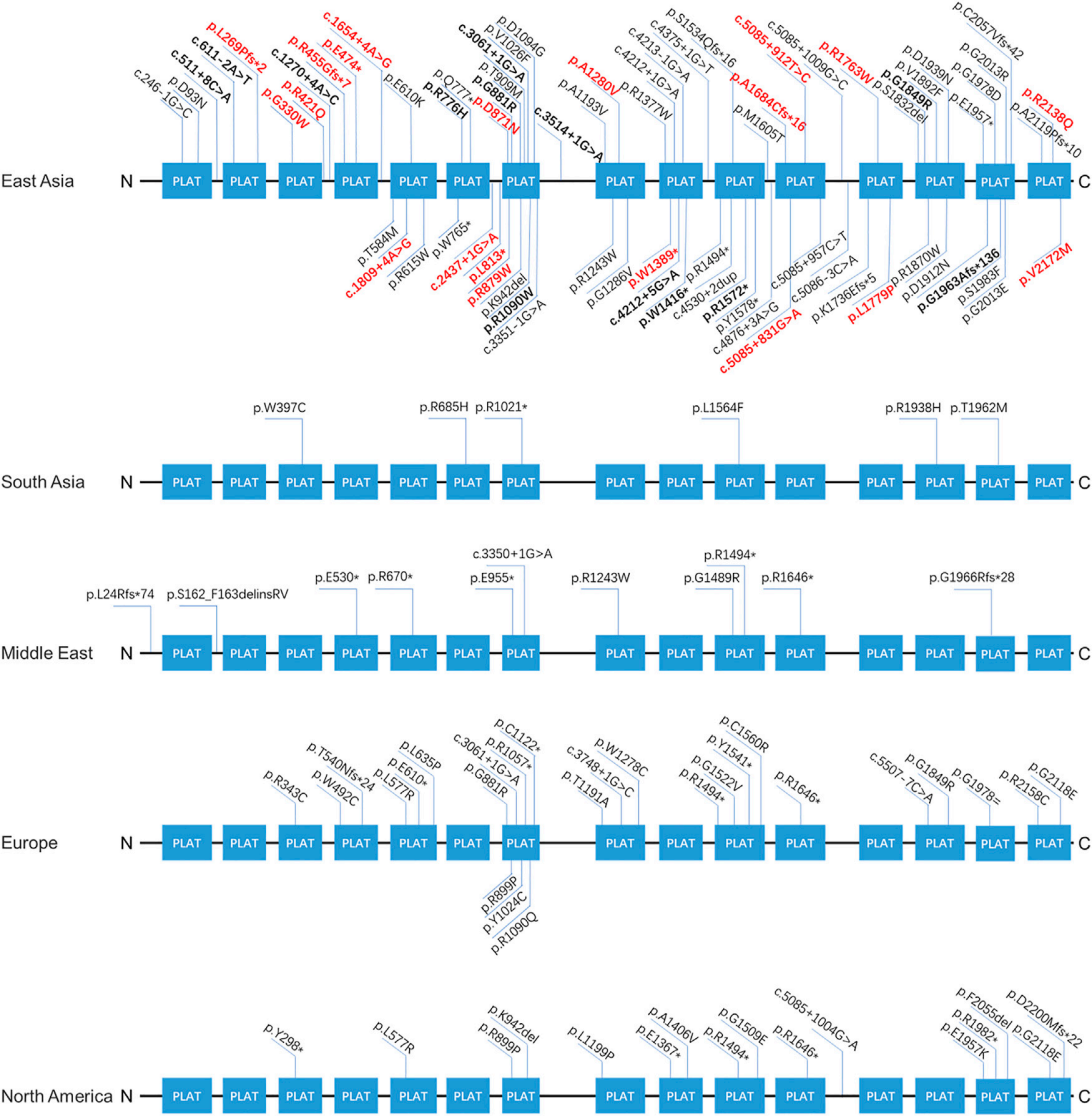
The spectra of *LOXHD1* variants in different populations. The *LOXHD1* gene here and its protein consists of 15 PLAT domains (NM_144612, NP_653213). All variants identified in this study are marked in bold, the novel variants are highlighted in red in the meantime.

Our findings expand the spectrum of variants seen in *LOXHD1*. Supplementary Table S3 summarizes the position, origin, and pathogenicity classification of the 124 *LOXHD1* variants filtered using the Human Gene Mutation Database (HGMD) Professional version (release 2021.02) and appraised through literature review to date, including the 20 novel variants identified in this study. The findings have also expanded the variant spectrum of *LOXHD1* in the Chinese deafness population from 28 to 51. Figure 3 shows the loci of *LOXHD1* variants seen in different populations on the schematic diagram of the protein domain structure. Among all the 13 reported variants, c.5888delG (p.G1963Afs*136), c.611–2A > T, c.3268C > T (p.R1090W), c.4247G > A (p.W1416*) and c.4212+5G > A (NM_144612.6) have only been reported in Chinese HL patients (Bai et al., 2020; Liu et al., 2020; Yu et al., 2021), c.1270+4A > C was reported in a Japanese family (Kim et al., 2021), and the other variants were reported mainly in European and American multi-ethnic populations (Sloan-Heggen et al., 2016; Zazo et al., 2017; Safka et al., 2020). Notably, both c.5888delG and c.611–2A > T had a relatively high recurrent frequency as 0.0862% (5/5,802) and 0.0517% (3/5,802) in our study, therefore, we speculate that they may be hot spot variants in the Chinese HL population.

This study has two minor limitations. First, most patients in our cohort did not present with multiple audiograms, leading to difficulties in estimating HL progression. We, therefore, had to rely on parental observation. Second, copy number variations in *LOXHD1* were not detected in this study; these should be investigated in further studies.

In conclusion, we evaluated the importance of *LOXHD1* variants in 2,901 Chinese patients with NSHL and investigated the clinical and genetic features of 21 patients. Our findings indicate that variants in *LOXHD1* represent a comparatively common cause of NSHL in the Chinese population. It is hoped that these findings will assist with diagnosis, management, and genetic counseling of NSHL patients, as well as suggesting directions for therapeutic development.

## Data Availability

The datasets presented in this study can be found in online repositories. The names of the repository/repositories and accession number(s) can be found in the article/Supplementary Material.

## Supplementary Material

Supplementary Figure S1 Scatter plots of hearing phenotypes of 21 patients after grouped by variant location and variant severity. Type1: grouped by variant location; Type2: grouped by variant severity.

Supplementary Figure S2 Proportions of different types of variation of *LOXHD1* both worldwide and in China. **(A)** Proportions of different types of variation of *LOXHD1* worldwide. Types of variation were broken down as missense, nonsense, frameshift, splice-site, intronic and synonymous. **(B)** Proportions of different types of variation of *LOXHD1* in China. Types of variation were broken down the same as those in Panel **(A)**.
**(C)**Stacked bar chart of different types of variation of *LOXHD1* in China vs. worldwide.

## Abbreviations

HL, hearing loss; NSHL, non-syndromic hearing loss; ARNSHL, autosomal recessive non-syndromic hearing loss; ACMG, American College of Medical Genetics and Genomics; AMP, Association for Molecular Pathology; CT, computed tomography; ASHA, American Speech-Language-Hearing Association.
