# Supplementary material for: Genetic Analysis of the LOXHD1 Gene in Chinese Patients With Non-Syndromic Hearing Loss
Source: Front Genet. 2022 May 27;13:825082. doi: 10.3389/fgene.2022.825082 (PMC9196635; doi:10.3389/fgene.2022.825082)
Supplement: Supplementary file 1 [file Table1.docx]

| ACTG1 | ADGRV1 | ALX3 | BSND | CABP2 | CCDC50 | CDH23 |
| --- | --- | --- | --- | --- | --- | --- |
| CEACAM16 | CHD7 | CIB2 | CLDN14 | CLPP | CLRN1 | COCH |
| COL11A1 | COL11A2 | COL1A1 | COL1A2 | COL2A1 | COL4A3 | COL4A4 |
| COL4A5 | COL4A6 | COL9A1 | COL9A2 | CRYM | DFNA5 | WHRN |
| DFNB59 | DIABLO | DIAPH1 | DIAPH3 | DSPP | ECM1 | EDN3 |
| EDNRB | ELMOD3 | ESPN | ESRRB | EYA1 | EYA4 | FGF3 |
| FGF8 | FGFR1 | FGFR3 | FLNA | FOXI1 | FREM1 | FXN |
| GATA3 | GIPC3 | GJB1 | GJB2 | GJB3 | GJB6 | GLYAT |
| GPSM2 | GRHL2 | GRXCR1 | HARS | HARS2 | HGF | HMX1 |
| HOXA2 | HSD17B4 | IL13 | ILDR1 | KARS | KCNE1 | KCNJ10 |
| KCNQ1 | KCNQ4 | KITLG | KRT9 | LAMA3 | LARS2 | LHFPL5 |
| LOXHD1 | LRTOMT | MARVELD2 | MIR96 | MITF | MPZ | MSRB3 |
| MYH14 | MYH9 | MYO15A | MYO1A | MYO1E | MYO3A | MYO6 |
| MYO7A | NDP | NDRG1 | NEFL | NELL2 | NF2 | OPA1 |
| OTOA | OTOF | OTOG | OTOGL | P2RX2 | PABPN1 | PAX3 |
| PCDH15 | PCDH9 | PDZD7 | PMP22 | PNPT1 | POLR1C | POLR1D |
| POU3F4 | POU4F3 | PROK2 | PROKR2 | PRPS1 | PTPN11 | PTPRQ |
| PTPRR | RDX | RPGR | SALL1 | SALL4 | SEC23A | SEMA3E |
| SERPINB6 | SIX1 | SIX5 | SLC17A8 | SLC19A2 | SLC26A4 | SLC26A5 |
| SMAD4 | SMPX | SNAI2 | SOX10 | STRC | TBC1D24 | TCIRG1 |
| TCOF1 | TECTA | TIMM8A | TJP2 | TMC1 | TMEM126A | TMIE |
| TMPRSS3 | TMPRSS4 | TNC | TPRN | TRIOBP | TRMU | TSPEAR |
| TYR | USH1C | USH1G | USH2A | WFS1 |  |  |

**Table S1 List of 159 deafness genes**
