# Supplementary figures and images for: Genetic Analysis of the LOXHD1 Gene in Chinese Patients With Non-Syndromic Hearing Loss
Source: Front Genet. 2022 May 27;13:825082. doi: 10.3389/fgene.2022.825082 (PMC9196635; doi:10.3389/fgene.2022.825082)

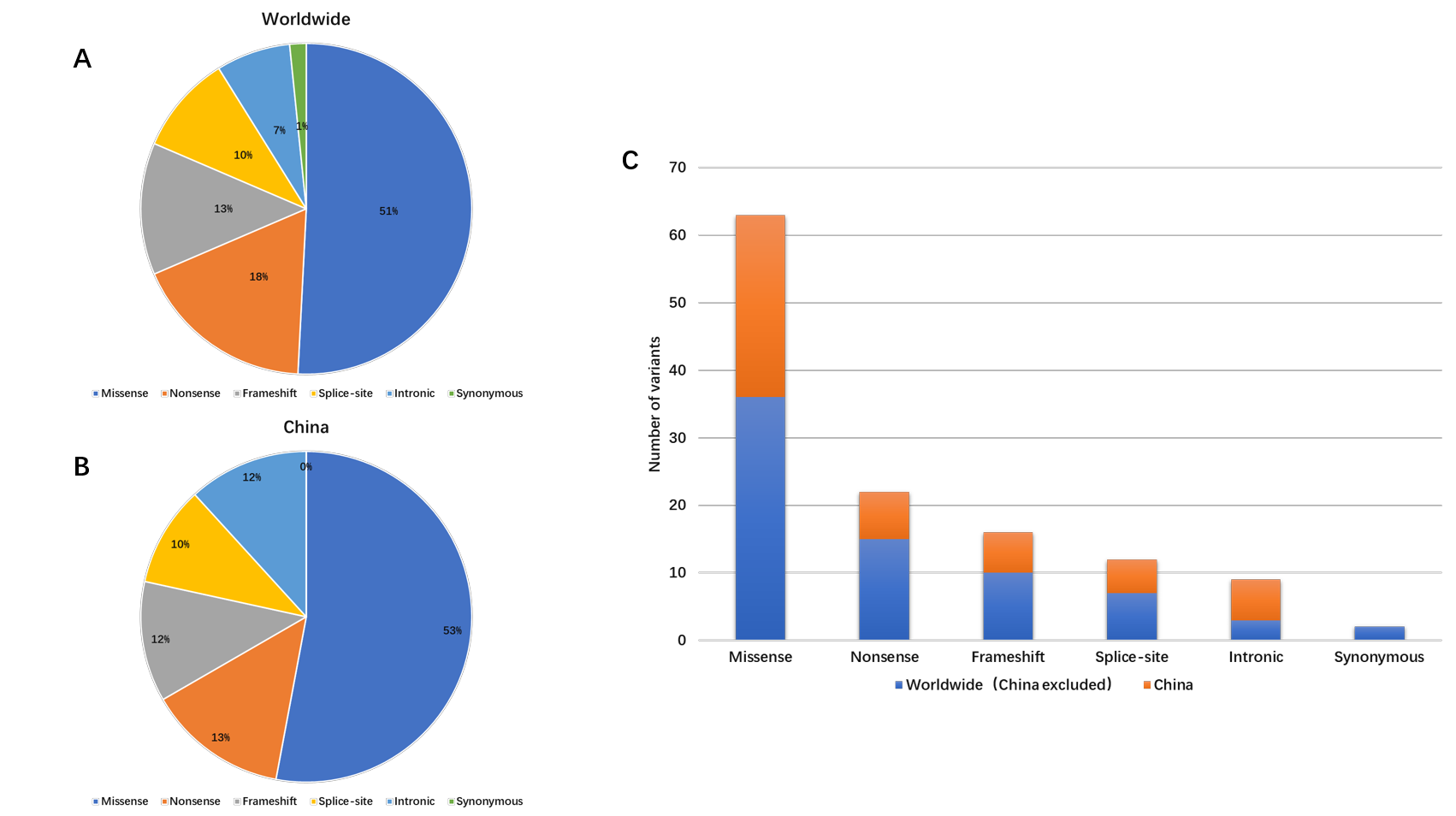

Supplement: Supplementary file 2 [file Image2.TIF]

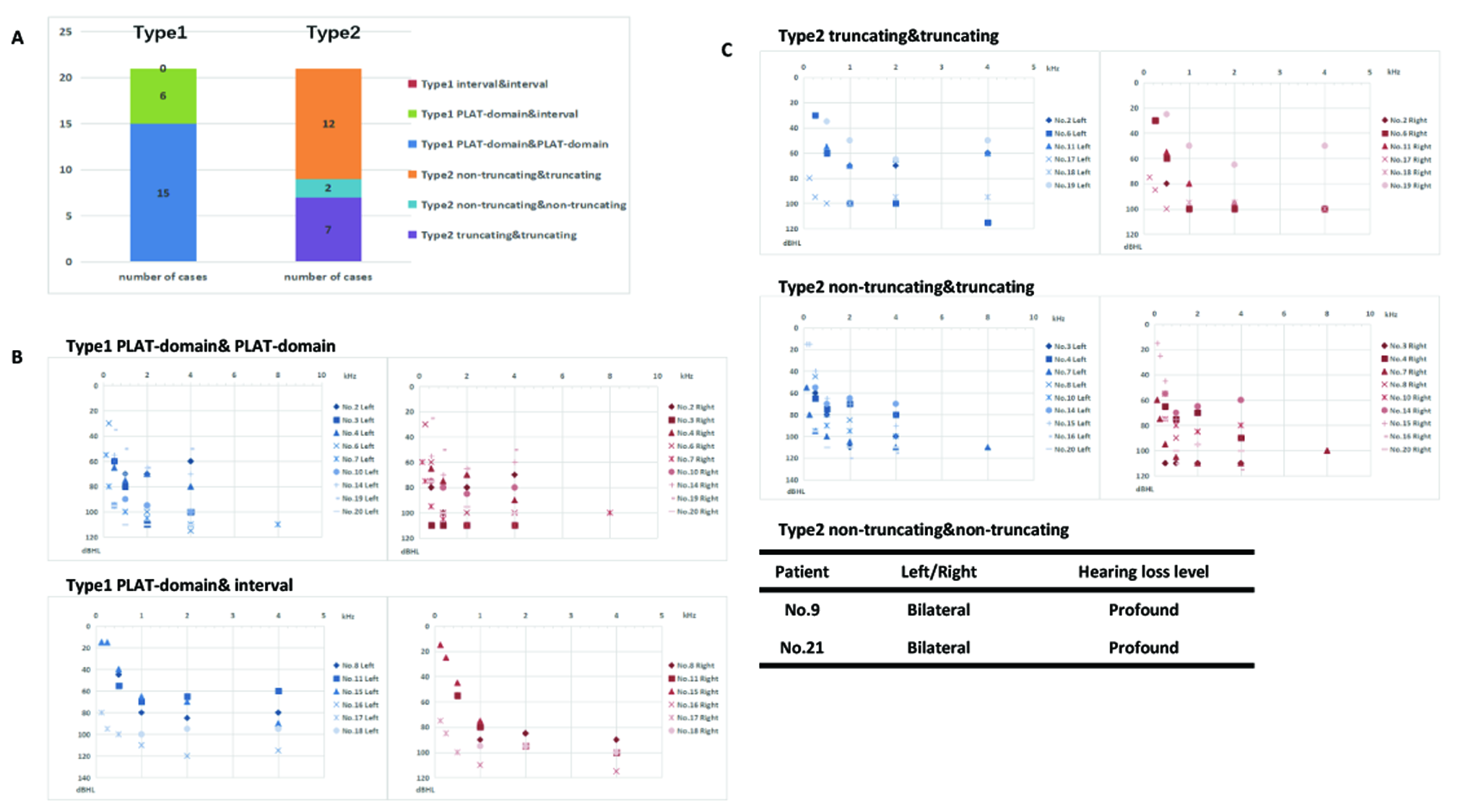

Supplement: Supplementary file 3 [file Image1.TIF]
