# Supplementary material for: Genetic Analysis of the LOXHD1 Gene in Chinese Patients With Non-Syndromic Hearing Loss
Source: Front Genet. 2022 May 27;13:825082. doi: 10.3389/fgene.2022.825082 (PMC9196635; doi:10.3389/fgene.2022.825082)
Supplement: Supplementary file 4 [file Table2.docx]

**Table S2 Summary of the candidate variants identified in the NGS test**

| **Case ID** | **Gene** | **Transcript** | **Nucleotide change** | **Amino acid change** | **hom/**  **het** | **Allele Frequency*** | **Pathogenicity** | **ACMG code** | **Inheritance mode** | **Origin of variant** | **Co-segregation** |
| --- | --- | --- | --- | --- | --- | --- | --- | --- | --- | --- | --- |
| 1 | **LOXHD1** | **NM_144612.6** | **c.2641G>A** | **p.(Gly881Arg)** | **het** | **-** | **Likely Pathogenic** | **PS1+PM2** | **AR** | **paternal** | **Yes** |
|  | **LOXHD1** | **NM_144612.6** | **c.1420G>T** | **p.(Glu474Ter)** | **het** | **-** | **Pathogenic** | **PVS1+PM2+PP3** | **AR** | **maternal** | **Yes** |
|  | HARS1 | NM_002109.6 | c.1256A>T | p.(Lys419Met) | het | 0.00109 | Uncertain significance | PM2+PP3 | AD/AR | - | - |
|  | HARS1 | NM_002109.6 | c.1255A>C | p.(Lys419Gln) | het | 0.00109 | Uncertain significance | PM2+BP4 | AD/AR | - | - |
| 2 | **LOXHD1** | **NM_144612.6** | **c.5888delG** | **p.(Gly1963AlafsTer136)** | **het** | **0.000183** | **Pathogenic** | **PVS1+PS1+PM2+PP3+PP5** | **AR** | **maternal** | **Yes** |
|  | **LOXHD1** | **NM_144612.6** | **c.4714C>T** | **p.(Arg1572Ter)** | **het** | **-** | **Pathogenic** | **PVS1+PS1+PP5+PM2+PP3** | **AR** | **paternal** | **Yes** |
| 3 | **LOXHD1** | **NM_144612.6** | **c.1362delG** | **p.(Arg455GlyfsTer7)** | **het** | **-** | **Pathogenic** | **PVS1+PM2+PP3** | **AR** | **maternal** | **Yes** |
|  | **LOXHD1** | **NM_144612.6** | **c.2641G>A** | **p.(Gly881Arg)** | **het** | **-** | **Likely Pathogenic** | **PS1+PM2** | **AR** | **paternal** | **Yes** |
| 4 | **LOXHD1** | **NM_144612.6** | **c.3061+1G>A** | **-** | **het** | **-** | **Pathogenic** | **PVS1+PM1+PP5+PM2+PP3** | **AR** | **paternal** | **Yes** |
|  | **LOXHD1** | **NM_144612.6** | **c.5336T>C** | **p.(Leu1779Pro)** | **het** | **-** | **Uncertain Significance** | **PM2+BP1+BP4** | **AR** | **maternal** | **Yes** |
| 5 | **LOXHD1** | **NM_144612.6** | **c.4247G>A** | **p.(Trp1416Ter)** | **het** | **-** | **Pathogenic** | **PVS1+PM1+PM2+PP3+PP5** | **AR** | **paternal** | **Yes** |
|  | **LOXHD1** | **NM_144612.6** | **c.6413G>A** | **p.(Arg2138Gln)** | **het** | **0.033** | **Uncertain Significance** | **PM2+PP3+BP1** | **AR** | **maternal** | **Yes** |
| 6 | **LOXHD1** | **NM_144612.6** | **c.4167G>A** | **p.(Trp1389Ter)** | **het** | **-** | **Pathogenic** | **PVS1+PM2+PP3** | **AR** | **maternal** | **Yes** |
|  | **LOXHD1** | **NM_144612.6** | **c.1809+4A>G** | **-** | **het** | **0.0000884** | **Uncertain Significance** | **PM2+BP4** | **AR** | **paternal** | **Yes** |
| 7 | **LOXHD1** | **NM_144612.6** | **c.611-2A>T** | **-** | **het** | **-** | **Pathogenic** | **PVS1+PM1+PM2+PP3** | **AR** | **-** | **-** |
|  | **LOXHD1** | **NM_001145472.2** | **c.1846T>C** | **p.(Cys616Arg)** | **het** | **-** | **Uncertain Significance** | **PM2+PP3+BP1** | **AR** | **maternal** | **-** |
| 8 | **LOXHD1** | **NM_144612.6** | **c.2438T>A** | **p.(Leu813Ter)** | **het** | **-** | **Likely Pathogenic** | **PVS1+PM2** | **AR** | **paternal** | **Yes** |
|  | **LOXHD1** | **NM_144612.6** | **c.2635C>T** | **p.(Arg879Trp)** | **het** | **-** | **Uncertain Significance** | **PM2+PP3+BP1** | **AR** | **maternal** | **Yes** |
|  | MYO15A | NM_016239.4 | c.3658G>A | p.(Gly1220Arg) | het | 0.0335 | Benign | PP2+BS1+BS2+BP4 | AR | paternal | Yes |
|  | MYO15A | NM_016239.4 | c.5557C>G | p.(His1853Asp) | het | 0.000111 | Uncertain Significance | PM1+PM2+PP2+BP4 | AR | maternal | Yes |
| 9 | **LOXHD1** | **NM_144612.6** | **c.6413G>A** | **p.(Arg2138Gln)** | **het** | **0.033** | **Uncertain Significance** | **PM2+PP3+BP1** | **AR** | **paternal** | **Yes** |
|  | **LOXHD1** | **NM_144612.6** | **c.2611G>A** | **p.(Asp871Asn)** | **het** | **0.0000932** | **Uncertain Significance** | **PM2+BP1** | **AR** | **maternal** | **Yes** |
| 10 | **LOXHD1** | **NM_144612.6** | **c.805dupC** | **p.(Leu269ProfsTer2)** | **het** | **-** | **Pathogenic** | **PVS1+PM2+PP3** | **AR** | **paternal** | **Yes** |
|  | **LOXHD1** | **NM_144612.6** | **c.5545G>A** | **p.(Gly1849Arg)** | **het** | **0.0000917** | **Uncertain Significance** | **PM1+PM2+PP3+BP1** | **AR** | **maternal** | **Yes** |
| 11 | **LOXHD1** | **NM_144612.6** | **c.2437+1G>A** | **-** | **het** | **-** | **Pathogenic** | **PVS1+PM2+PP)** | **AR** | **paternal** | **Yes** |
|  | **LOXHD1** | **NM_144612.6** | **c.4247G>A** | **p.(Trp1416Ter)** | **het** | **-** | **Pathogenic** | **PVS1+PM1+PM2+PP3+PP5** | **AR** | **maternal** | **Yes** |
| 12 | **LOXHD1** | **NM_144612.6** | **c.1420G>T** | **p.(Glu474Ter)** | **het** | **-** | **Pathogenic** | **PVS1+PM2+PP3** | **AR** | **paternal** | **Yes** |
|  | **LOXHD1** | **NM_001145472.2** | **c.879+5G>A** | **-** | **het** | **-** | **Uncertain Significance** | **PM1+PM2+PP5** | **AR** | **maternal** | **Yes** |
| 13 | **LOXHD1** | **NM_144612.6** | **c.611-2A>T** | **-** | **het** | **-** | **Pathogenic** | **PVS1+PM1+PM2+PP3** | **AR** | **paternal** | **Yes** |
|  | **LOXHD1** | **NM_144612.6** | **c.988G>T** | **p.(Gly330Trp)** | **het** | **-** | **Uncertain Significance** | **PM2+PP3+BP1** | **AR** | **maternal** | **Yes** |
| 14 | **LOXHD1** | **NM_144612.6** | **c.3839C>T** | **p.(Ala1280Val)** | **het** | **-** | **Uncertain Significance** | **PM2+BP1+BP4** | **AR** | **paternal** | **Yes** |
|  | **LOXHD1** | **NM_144612.6** | **c.5888delG** | **p.(Gly1963AlafsTer136)** | **het** | **0.000183** | **Pathogenic** | **PVS1+PS1+PM2+PP3+PP5** | **AR** | **maternal** | **Yes** |
| 15 | **LOXHD1** | **NM_001145472.2** | **c.1765G>A** | **p.(Gly589Arg)** | **het** | **-** | **Uncertain Significance** | **PM2+PP3+BP1** | **AR** | **paternal** | **Yes** |
|  | **LOXHD1** | **NM_144612.6** | **c.1270+4A>C** | **-** | **het** | **-** | **Uncertain Significance** | **PM1+PM2+BP4** | **AR** | **maternal** | **Yes** |
| 16 | **LOXHD1** | **NM_144612.6** | **c.511+8C>A** | **-** | **het** | **0.000551** | **Uncertain Significance** | **PM2+BP4+BP6** | **AR** | **paternal** | **Yes** |
|  | **LOXHD1** | **NM_144612.6** | **c.5287C>T** | **p.(Arg1763Trp)** | **het** | **-** | **Uncertain Significance** | **PM2+PP3+BP1** | **AR** | **maternal** | **Yes** |
| 17 | **LOXHD1** | **NM_144612.6** | **c.5888delG** | **p.(Gly1963AlafsTer136)** | **het** | **0.000183** | **Pathogenic** | **PVS1+PS1+PM2+PP3+PP5** | **AR** | **-** | **-** |
|  | **LOXHD1** | **NM_144612.6** | **c.3514+1G>A** | **-** | **het** | **-** | **Pathogenic** | **PVS1+PM2+PP3+PP5** | **AR** | **-** | **-** |
| 18 | **LOXHD1** | **NM_144612.6** | **c.1654+4A>G** | **-** | **het** | **-** | **Uncertain Significance** | **PM2** | **AR** | **paternal** | **Yes** |
|  | **LOXHD1** | **NM_144612.6** | **c.611-2A>T** | **-** | **het** | **-** | **Pathogenic** | **PVS1+PM1+PM2+PP3** | **AR** | **maternal** | **Yes** |
| 19 | **LOXHD1** | **NM_144612.6** | **c.5888delG** | **p.(Gly1963AlafsTer136)** | **hom** | **0.000183** | **Pathogenic** | **PVS1+PS1+PM2+PP3+PP5** | **AR** | **paternal/**  **maternal** | **Yes** |
|  | **LOXHD1** | **NM_144612.6** | **c.1262G>A** | **p.(Arg421Gln)** | **het** | **0.000619** | **Uncertain Significance** | **PM2+BP1+BP4** | **AR** | **paternal** | **-** |
| 20 | **LOXHD1** | **NM_144612.6** | **c.3268C>T** | **p.(Arg1090Trp)** | **het** | **-** | **Uncertain Significance** | **PM1+PM2+PP3+BP1** | **AR** | **paternal** | **Yes** |
|  | **LOXHD1** | **NM_001145472.2** | **c.1716_1717insT** | **-** | **het** | **-** | **Pathogenic** | **PVS1+PM2+PP3** | **AR** | **maternal** | **Yes** |
| 21 | **LOXHD1** | **NM_144612.6** | **c.2327G>A** | **p.(Arg776His)** | **het** | **0.000265** | **Uncertain Significance** | **PM2+BP1+BP4** | **AR** | **paternal** | **Yes** |
|  | **LOXHD1** | **NM_001145473.2** | **c.1417G>A** | **p.(Val473Met)** | **het** | **-** | **Uncertain Significance** | **PVS1+PM2+PP3** | **AR** | **maternal** | **Yes** |

*: Allele Frequency in East Asian reported by gnomAD; -: no data; Bold, back indicates the candidate pathogenic variants; Red indicates compound heterozygous variants with uncertain significance.
