# Supplementary material for: Genetic Analysis of the LOXHD1 Gene in Chinese Patients With Non-Syndromic Hearing Loss
Source: Front Genet. 2022 May 27;13:825082. doi: 10.3389/fgene.2022.825082 (PMC9196635; doi:10.3389/fgene.2022.825082)
Supplement: Supplementary file 5 [file Table3.docx]

**Table 3. All variants of *LOXHD1* reported worldwide(**[**NM_144612**](https://www.ncbi.nlm.nih.gov/nuccore/NM_144612)**.6).**

| **No.** | **Location** | **Variant** | **AA change** | **Exon/Intron** | **domain** | **Ethnicity** | **Pathogenecity** | **Reference** |
| --- | --- | --- | --- | --- | --- | --- | --- | --- |
| 1 | chr18:44236926 | c.71del | p.Leu24ArgfsTer74 | Exon1/40 | --- | Turkish | Pathogenic  (PVS1+PM2+PP3) | Atik T, *et al*.,2015 |
| 2 | chr18:44229229 | c.134A>C | p.Tyr45Ser | Exon2/40 | PLAT1 | Chinese | Uncertain Significance  (PM2+PP3) | Jin X, *et al*.,2022 |
| 3 | chr18:44222000 | c.246-1G>C | --- | Intron2/39 | PLAT1 | Japanese | Pathogenic  (PVS1+PM2+PP3) | [Maekawa](https://pubmed.ncbi.nlm.nih.gov/?sort=date&size=20&term=Maekawa+K&cauthor_id=31547530) K, *et al*.,2019 |
| 4 | chr18:44221968 | c.277G>A | p.Asp93Asn | Exon3/40 | PLAT1 | Chinese | Likely Pathogenic  (PM1+PM2+PP5+BP1) | Bai X, *et al*.,2020 |
| 5 | chr18:44219648 | c.442A>T | p.Lys148Ter | Exon4/40 | PLAT1 | ---  Czech | Pathogenic  (PVS1+PP5+PM2+PP3) | Posey JE, *et al.*, 2017  Safka Brozkova D, *et al*.,2020 |
| 6 | chr18:44219603 | c.486_487delCTinsGG | p.Ser162_Phe163delinsArgVal | Exon4/40 | interval  (PLAT1-2) | Arab | Uncertain Significance  (PM2+PP3) | Abouelhoda M, *et al.*, 2016 |
| 7 | chr18:44219571 | c.511+8C>A | --- | Intron4/39 | interval  (PLAT1-2) | Chinese | Uncertain Significance  (PM2+BP4+BP6) | Present study |
| 8 | chr18:44190889 | c.611-2A>T | --- | Intron5/39 | PLAT2 | Chinese | Pathogenic  (PVS1+PM1+PM2+PP3) | Present study  Bai X, *et al*.,2020 |
| 9 | chr18:44184147 | c.805dupC | p.Leu269ProfsTer2 | Exon7/40 | PLAT2 | Chinese | Pathogenic  (PVS1+PM2+PP3) | Present study |
| 10 | chr18:44181420 | c.894T>G | p.Tyr298Ter | Exon8/40 | PLAT3 | --- | Pathogenic  (PVS1+PM2+PP3+PP5) | Sloan-Heggen CM, *et al*.,2016 |
| 11 | chr18:44181326 | c.988G>T | p.Gly330Trp | Exon8/40 | PLAT3 | Chinese | Uncertain Significance  (PM2+PP3+BP1) | Present study |
| 12 | chr18:44181287 | c.1027C>T | p.Arg343Cys | Exon8/40 | PLAT3 | Czech | Uncertain Significance  (PM2+PP3+BP1) | Safka Brozkova D, *et al*.,2020 |
| 13 | chr18:44174373 | c.1191G>T | p.Trp397Cys | Exon9/40 | PLAT3 | Indian | Uncertain Significance  (PM2+PP3+BP1) | Chakrabarty S, *et al*.,2021 |
| 14 | chr18:44174302 | c.1262G>A | p.Arg421Gln | Exon9/40 | interval  (PLAT3-4) | Chinese | Uncertain Significance  (PM2+BP1+BP4) | Present study |
| 15 | chr18:44174290 | c.1270+4A>C | --- | Intron9/39 | interval  (PLAT3-4) | Chinese  Japanese | Uncertain Significance  (PM1+PM2+BP4) | Present study  [Maekawa](https://pubmed.ncbi.nlm.nih.gov/?sort=date&size=20&term=Maekawa+K&cauthor_id=31547530) K, *et al*.,2019 |
| 16 | chr18:44173632 | c.1362delG | p.Arg455GlyfsTer7 | Exon10/40 | PLAT4 | Chinese | Pathogenic  (PVS1+PM2+PP3) | Present study |
| 17 | chr18:44173574 | c.1420G>T | p.Glu474Ter | Exon10/40 | PLAT4 | Chinese | Pathogenic  (PVS1+PM2+PP3) | Present study |
| 18 | chr18:44172503 | c.1476G>T | p.Trp492Cys | Exon11/40 | PLAT4 | Czech | Uncertain Significance  (PM2+PP3+BP1) | Safka Brozkova D, *et al*.,2020 |
| 19 | chr18:44171962 | c.1588G>T | p.Glu530Ter | Exon12/40 | PLAT4 | Qatari | Pathogenic  (PVS1+PM2+PP5+PP3) | Abouelhoda M, *et al.*, 2016 |
| 20 | chr18:44171932 | c.1618dup | p.Thr540AsnfsTer24 | Exon12/40 | PLAT4 | Dutch | Pathogenic  (PVS1+PM2 +PP3) | Wesdorp M, *et al.*, 2018 |
| 21 | chr18:44171892 | c.1654+4A>G | --- | Intron12/39 | interval  (PLAT4-5) | Chinese | Uncertain Significance  (PM2) | Present study |
| 22 | chr18:44159672 | c.1730T>G | p.Leu577Arg | Exon13/40 | PLAT5 | Dutch  --- | Uncertain Significance  (PM1+PM2+PP3+BP1) | Wesdorp M, *et al.*, 2018  Sloan-Heggen CM, et al.,2016 |
| 23 | chr18:44159651 | c.1751C>T | p.Thr584Met | Exon13/40 | PLAT5 | Chinese | Uncertain Significance  (PM1+PM2+PP3+BP1) | Hu S, *et al*., 2018 |
| 24 | chr18:44159589 | c.1809+4A>G | --- | Intron13/39 | PLAT5 | Chinese | Uncertain Significance  (PM2+BP4) | Present study |
| 25 | chr18:44157812 | c.1828G>A | p.Glu610Lys | Exon14/40 | PLAT5 | Dutch  Chinese | Likely Pathogenic  (PM1+PM2+PP5+PP3+BP1) | Wesdorp M, *et al*.,2018  [Zhang](https://pubmed.ncbi.nlm.nih.gov/?sort=date&size=20&term=Zhang+C&cauthor_id=31709873) C, *et al*., 2019 |
| 26 | chr18:44157797 | c.1843C>T | p.Arg615Trp | Exon14/40 | PLAT5 | Chinese | Uncertain Significance  (PM1+ PM2+BP1+BP4) | Zhou Y, *et al*., 2016 |
| 27 | chr18:44157736 | c.1904T>C | p.Leu635Pro | Exon14/40 | PLAT5 | Dutch | Likely Pathogenic  (PM1+PM2+PP3+PP5) | Wesdorp M, *et al*.,2018 |
| 28 | chr18:44152088 | c.2008C>T | p.Arg670Ter | Exon15/40 | PLAT5 | Iranian | Pathogenic  (PVS1+PP5+PM2+PP3) | Grillet N, *et al*.,2009 |
| 29 | chr18:44149595 | c.2054G>A | p.Arg685His | Exon16/40 | PLAT6 | Indian | Uncertain Significance  (PM2+BP1+BP4) | Chakrabarty S, *et al*.,2021 |
| 30 | chr18:44146362 | c.2295G>A | p.Trp765Ter | Exon17/40 | PLAT6 | Chinese | Pathogenic  (PVS1+PM2+PP3) | Jin X, *et al*.,2022 |
| 31 | chr18:44146330 | c.2327G>A | p.Arg776His | Exon17/40 | PLAT6 | Chinese | Uncertain Significance  (PM2+BP1+BP4) | Present study |
| 32 | chr18:44146328 | c.2329C>T | p.Gln777Ter | Exon17/40 | PLAT6 | Chinese | Pathogenic  (PVS1+PM2+PP5+PP3) | Bai X, *et al*.,2020 |
| 33 | chr18:44146219 | c.2437+1G>A | --- | Intron17/39 | interval  (PLAT6-7) | Chinese | Pathogenic  (PVS1+PM2+PP3) | Present study |
| 34 | chr18:44143188 | c.2438T>A | p.Leu813Ter | Exon18/40 | interval  (PLAT6-7) | Chinese | Likely Pathogenic  (PVS1+PM2) | Present study |
| 35 | chr18:44140496 | c.2611G>A | p.Asp871Asn | Exon19/40 | PLAT7 | Chinese | Uncertain Significance  (PM2+BP1) | Present study |
| 36 | chr18:44140472 | c.2635C>T | p.Arg879Trp | Exon19/40 | PLAT7 | Chinese | Uncertain Significance  (PM2+PP3+BP1) | Present study |
| 37 | chr18:44140466 | c.2641G>A | p.Gly881Arg | Exon19/40 | PLAT7 | Chinese  Dutch | Likely Pathogenic  (PS1+PM2) | Present study  Wesdorp M, *et al*.,2018 |
| 38 | chr18:44140411 | c.2696G>C | p.Arg899Pro | Exon19/40 | PLAT7 | Czech  Dutch  --- | Uncertain Significance  (PM2+PP3+BP1) | Safka Brozkova D, *et al*.,2020  Wesdorp M, *et al*.,2018  Sloan-Heggen CM, *et al*.,2016 |
| 39 | chr18:44140381 | c.2726C>T | p.Thr909Met | Exon19/40 | PLAT7 | Japanese | Uncertain Significance  (PM2+BP1+BP4) | [Maekawa](https://pubmed.ncbi.nlm.nih.gov/?sort=date&size=20&term=Maekawa+K&cauthor_id=31547530) K, *et al*.,2019 |
| 40 | chr18:44140280-44140282 | c.2825-2827delAGA | p.Lys942del | Exon19/40 | PLAT7 | ---  Chinese | Uncertain Significance  (PS1+PP3+BS1) | Sloan-Heggen CM, *et al*.,2016  [Zhang](https://pubmed.ncbi.nlm.nih.gov/?sort=date&size=20&term=Zhang+C&cauthor_id=31709873) C, *et al*., 2019 |
| 41 | chr18:44140244 | c.2863G>T | p.Glu955Ter | Exon19/40 | PLAT7 | Turkish | Pathogenic  (PVS1+PP5+PM2+PP3) | Diaz-Horta O, *et al*., 2012 |
| 42 | chr18:44140046 | c.3061C>T | p.Arg1021Ter | Exon19/40 | PLAT7 | Dutch | Pathogenic  (PVS1+PP5+PM2+PP3) | Zazo Seco C, *et al*.,2017 |
| 43 | chr18:44140045 | c.3061+1G>A | --- | Intron19/39 | PLAT7 | Chinese  Dutch | Pathogenic  (PVS1+PM1+PP5+PM2+PP3) | Present study  Zazo Seco C, *et al*.,2017 |
| 44 | chr18:44139556 | c.3071A>G | p.Tyr1024Cys | Exon20/40 | PLAT7 | Italian | Uncertain Significance  (PM2+PP3+BP1) | Morgan A, *et al*.,2018 |
| 45 | chr18:44139551 | c.3076G>T | p.Val1026Phe | Exon20/40 | PLAT7 | Japanese | Uncertain Significance  (PM2+PP3+BP1) | Sakuma N, *et al*.,2016 |
| 46 | chr18:44139458 | c.3169C>T | p.Arg1057Ter | Exon20/40 | PLAT7 | Dutch | Pathogenic  (PVS1+PP5+PM2+PP3) | Wesdorp M, *et al*.,2018 |
| 47 | chr18-44137401 | c.3268C>T | p.Arg1090Trp | Exon21/40 | PLAT7 | Chinese | Uncertain Significance  (PM1+PM2+PP3+BP1) | Present study  Liu XW, *et al*.,2020 |
| 48 | chr18:44137400 | c.3269G>A | p.Arg1090Gln | Exon21/40 | PLAT7 | Icelander/[British](javascript:;) | Benign  (PP3+BS1+BS2+BP1) | Ivarsdottir EV, *et al*.,2021 |
| 49 | chr18:44137388 | c.3281A>G | p.Asp1094Gly | Exon21/40 | PLAT7 | Chinese | Uncertain Significance  (PM2+PP3+BP1) | Zhou Y, *et al*., 2016 |
| 50 | chr18:44137318 | c.3350+1G>A | --- | Intron21/39 | PLAT7 | Egyptian | Pathogenic  (PVS1+PM2+PP3+PP5) | Budde BS, *et al*., 2020 |
| 51 | chr18:44127022 | c.3351-1G>A | --- | Intron21/39 | PLAT7 | Chinese | Pathogenic  (PVS1+PM2+PP3+PP5) | Yu S, *et al*.,2021 |
| 52 | chr18:44127006 | c.3366C>A | p.Cys1122Ter | Exon22/40 | PLAT7 | Czech | Pathogenic  (PVS1+PM2+PP3) | Safka Brozkova D, *et al*.,2020 |
| 53 | chr18:44127001 | c.3371G>A | p.Arg1124His | Exon22/40 | PLAT7 | Cameroonian | Uncertain Significance  (PM2+PP3+BP1) | Lebeko K, *et al*.,2016 |
| 54 | chr18:44126857 | c.3514+1G>A | --- | Intron22/39 | interval  (PLAT7-8) | Chinese | Pathogenic  (PVS1+PM2+PP3+PP5) | Present study |
| 55 | chr18:44125328 | c.3571A>G | p.Thr1191Ala | Exon23/40 | PLAT8 | Spanish | Uncertain Significance  (PM2+PP3+BP1) | Cabanillas R, *et al*.,2018 |
| 56 | chr18:44125321 | c.3578C>T | p.Ala1193Val | Exon23/40 | PLAT8 | Japanese | Uncertain Significance  (PM2+PP3+BP1) | [Maekawa](https://pubmed.ncbi.nlm.nih.gov/?sort=date&size=20&term=Maekawa+K&cauthor_id=31547530) K, *et al*.,2019 |
| 57 | chr18:44125303 | c.3596T>C | p.Leu1199Pro | Exon23/40 | PLAT8 | --- | Uncertain Significance  (PM2+PP3+BP1 | Sloan-Heggen CM, *et al*.,2016 |
| 58 | chr18:44122711 | c.3727C>T | p.Arg1243Trp | Exon24/40 | PLAT8 | Korean  Egyptian | Uncertain Significance  (PM2+PP3+BP1) | Kim BJ, *et al*.,2021  Budde BS, *et al*., 2020 |
| 59 | chr18:44122689 | c.3748+1G>C | --- | Intron24/39 | PLAT8 | Dutch | Pathogenic  (PVS1+PM2+PP3) | Wesdorp M, *et al*.,2018 |
| 60 | chr18:44121818 | c.3834G>C | p.Trp1278Cys | Exon25/40 | PLAT8 | Dutch | Uncertain Significance  (PM2+PP3+BP1) | Wesdorp M, *et al*.,2018 |
| 61 | chr18:44121813 | c.3839C>T | p.Ala1280Val | Exon25/40 | PLAT8 | Chinese | Uncertain Significance  (PM2+BP1+BP4) | Present study |
| 62 | chr18:44121795 | c.3857G>T | p.Gly1286Val | Exon25/40 | PLAT8 | Japanese | Uncertain Significance  (PM2+PP3+BP1) | [Maekawa](https://pubmed.ncbi.nlm.nih.gov/?sort=date&size=20&term=Maekawa+K&cauthor_id=31547530) K, *et al*.,2019 |
| 63 | chr18:44118235 | c.3979T>A | p.Phe1327Ile | Exon26/40 | PLAT9 | Cameroonian | c.3979T>A | Lebeko K, *et al*.,2016 |
| 64 | chr18:44114411 | c.4099G>T | p.Glu1367Ter | Exon27/40 | PLAT9 | --- | Pathogenic  (PVS1+PP5+PM2+PP3) | Sloan-Heggen CM, *et al*.,2016 |
| 65 | chr18:44114381 | c.4129C>T | p.Arg1377Trp | Exon27/40 | PLAT9 | Chinese | Uncertain Significance  (PM2+PP3+BP1) | Liu XW, *et al*.,2020 |
| 66 | chr18:44114343 | c.4167G>A | p.Trp1389Ter | Exon27/40 | PLAT9 | Chinese | Pathogenic  (PVS1+PM2+PP3) | Present study |
| 67 | chr18:44114297 | c.4212+1G>A | --- | Intron27/39 | PLAT9 | Japanese  Czech | Pathogenic  (PVS1+PP5+PM2+PP3) | Mori K, *et al*.,2015  Plevova P, *et al*.,2017 |
| 68 | chr18:44114293 | c.879+5G>A  (NM_001145472.2)  [c.4212+5G>A  (NM_144612.6)] | --- | Intron9/23  [Intron27/39] | PLAT9 | Chinese | Uncertain Significance  (PM1+PM2+PP5) | Present study  Yu S, *et al*.,2021 |
| 69 | chr18:44113288 | c.4213-1G>A | --- | Intron27/39 | PLAT9 | Japanese | Pathogenic  (PVS1+PM2+PP3) | [Maekawa](https://pubmed.ncbi.nlm.nih.gov/?sort=date&size=20&term=Maekawa+K&cauthor_id=31547530) K, *et al*.,2019 |
| 70 | chr18:44113283 | c.4217C>T | p.Ala1406Val | Exon28/40 | PLAT9 | --- | Benign  (BS1+BS2+BP1) | Sloan-Heggen CM, *et al*.,2016 |
| 71 | chr18:44113253 | c.4247G>A | p.Trp1416Ter | Exon28/40 | PLAT9 | Chinese | Pathogenic  (PVS1+PM1+PM2+PP3+PP5) | Present study  Yu S, *et al*.,2021 |
| 72 | chr18:44113124 | c.4375+1G>T | ---- | Intron28/39 | interval  (PLAT9-10) | Japanese | Pathogenic  (PVS1+PM2+PP3+PP5) | Sakuma N, *et al*.,2016 |
| 73 | chr18:44109205 | c.4465G>C | p.Gly1489Arg | Exon29/40 | PLAT10 | Egyptian | Likely Pathogenic  (PS1+PM1+PM2+PP3+BP1) | Budde BS, *et al*., 2020 |
| 74 | chr18:44109190 | c.4480C>T | p.Arg1494Ter | Exon29/40 | PLAT10 | Czech  ---  Turkish  Caucasian  Japanese  Argentinean | Pathogenic  (PVS1+PP5+PM2+PP3) | Safka Brozkova D, *et al*.,2020  Sloan-Heggen CM, *et al*.,2016  Diaz-Horta O, *et al*., 2012  Eppsteiner RW, *et al*., 2012  Mori K, *et al*.,2015  Buonfiglio PI, *et al*.,2022 |
| 75 | chr18:44109144 | c.4526G>A | p.Gly1509Glu | Exon29/40 | PLAT10 | ---  Caucasian | Uncertain Significance  (PM1+BA1+BP1) | Sloan-Heggen CM, *et al*.,2016  Eppsteiner RW, *et al*., 2012 |
| 76 | chr18:44109138 | c.4530+2dup | --- | Intron29/39 | PLAT10 | Korean | Uncertain Significance  (PM2+PP3) | Kim BJ, *et al*.,2021 |
| 77 | chr18:44104846 | c.4565G>T | p.Gly1522Val | Exon30/40 | PLAT10 | Czech | Uncertain Significance  (PM1+PM2+ PP3+ BP1) | Safka Brozkova D, *et al*.,2020 |
| 78 | chr18:44104810 | c.4600_4601del | p.Ser1534GlnfsTer 16 | Exon30/40 | PLAT10 | Korean | Likely Pathogenic  (PVS1+PM2) | Kim BJ, *et al*.,2021 |
| 79 | chr18:44104788 | c.4623C>G | p.Tyr1541Ter | Exon30/40 | PLAT10 | Czech | Pathogenic  (PVS1+PM2+PP3) | Plevova P, *et al*.,2017 |
| 80 | chr18:44104733 | c.4678T>C | p.Cys1560Arg | Exon30/40 | PLAT10 | Dutch | Uncertain Significance  (PM1+PM2+ PP3+ BP1) | Wesdorp M, *et al*.,2018 |
| 81 | chr18:44104721 | c.4690C>T | p.Leu1564Phe | Exon30/40 | PLAT10 | Indian | Uncertain Significance  (PM1+PM2+ PP3+ BP1) | Chakrabarty S, *et al*.,2021 |
| 82 | chr18:44104697 | c.4714C>T | p.Arg1572Ter | Exon30/40 | PLAT10 | Chinese  Ashkenazi Jewish | Pathogenic  (PVS1+PS1+PP5+PM2+PP3) | Present study  Edvardson S, *et al*.,2011 |
| 83 | chr18:44104677 | c.4734C>G | p.Tyr1578Ter | Exon30/40 | PLAT10 | Japanese | Pathogenic  (PVS1+PM2+PP3) | [Maekawa](https://pubmed.ncbi.nlm.nih.gov/?sort=date&size=20&term=Maekawa+K&cauthor_id=31547530) K, *et al*.,2019 |
| 84 | chr18:44104491 | c.4814T>C | p.Met1605Thr | Exon31/40 | interval  (PLAT10-11) | Chinese | Uncertain Significance  (PM2+ BP1+ BP4) | Yu S, *et al*.,2021 |
| 85 | chr18:44104426 | c.4876+3A>G | --- | Intron31/39 | interval  (PLAT10-11) | Chinese | Uncertain Significance  (PM2+ PP5+ BP4) | Bai X, *et al*.,2020 |
| 86 | chr18:44102213 | c.4936C>T | p.Arg1646Ter | Exon32/40 | PLAT11 | Czech  Iranian  --- | Pathogenic  (PVS1+PP5+PM2+PP3) | Safka Brozkova D, *et al*.,2020  Kannan-Sundhari A, *et al*.,2020  Sloan-Heggen CM, *et al*.,2016 |
| 87 | chr18:44102100 | c.1716_1717insT  (NM_001145472.2)  [c.5049_5050insT  (NM_144612.6)] | p.Ala573CysfsTer16  [p.Ala1684CysfsTer16] | Exon14/24  [Exon32/40] | PLAT11 | Chinese | Pathogenic  (PVS1+PM2+PP3) | Present study |
| 88 | chr18:44101233 | c.1765G>A  (NM_001145472.2)  [c.5085+831G>A  (NM_144612.6)] | p.Gly589Arg | Exon15/24  [Intron32/39] | PLAT11 | Chinese | Uncertain Significance  (PM2+PP3+BP1) | Present study |
| 89 | chr18:44101152 | c.1846T>C  (NM_001145472.2)  [c.5085+912T>C  (NM_144612.6)] | p.Csy616Arg | Exon15/24  [Intron32/39] | PLAT11 | Chinese | Uncertain Significance  (PM2+PP3+BP1) | Present study |
| 90 | chr18:44101107 | c.1891C>T  (NM_001145472.2)  [c.5085+957C>T  (NM_144612.6)] | p.Arg631Cys | Exon15/24  [Intron32/39] | PLAT11 | Chinese | Uncertain Significance  PM2+PP3+PP5+BP1 | Yu S, *et al*.,2021 |
| 91 | chr18:44101060 | c.1938G>A  (NM_001145472.2)  [c.5085+1004G>A  (NM_144612.6)] | p.Lys646= | Exon15/24  [Intron32/39] | interval  (PLAT11-12) | --- | Likely Pathogenic  (PVS1+PM2) | Sloan-Heggen CM, *et al*.,2016 |
| 92 | chr18:44101055 | c.1938+5G>C  (NM_001145472.2)  c.5085+1009G>C  (NM_144612.6)] | --- | Intron32/39 | interval  (PLAT11-12) | Korean | Uncertain Significance  (PM2+BP4) | Kim BJ, *et al*.,2021 |
| 93 | chr18:44098222 | c.5086-3C>A | --- | Intron32/39 | interval  (PLAT11-12) | Japanese | c.5086-3C>A | [Maekawa](https://pubmed.ncbi.nlm.nih.gov/?sort=date&size=20&term=Maekawa+K&cauthor_id=31547530) K, *et al*.,2019 |
| 94 | chr18:44098101 | c.5204dup | p.Lys1736GlufsTer5 | Exon33/40 | PLAT12 | Korean | Pathogenic  (PVS1+PM2+PP3) | Kim BJ, *et al*.,2021 |
| 95 | chr18:44089705 | c.5287C>T | p.Arg1763Trp | Exon34/40 | PLAT12 | Chinese | Uncertain Significance  (PM2+PP3+BP1) | Present study |
| 96 | chr18:44087671 | c.5336T>C | p.Leu1779Pro | Exon35/40 | PLAT12 | Chinese | Uncertain Significance  (PM2+BP1+BP4) | Present study |
| 97 | chr18:44087511 | c.5494_5496del | p.Ser1832del | Exon35/40 | PLAT13 | Korean | Likely Pathogenic  (PM1+PM2+PM4+PP3) | Kim BJ, *et al*.,2021 |
| 98 | chr18:44085993 | c.5507-7C>A | --- | Intron35/39 | PLAT13 | Czech | Uncertain Significance  (PM2+BP4) | Safka Brozkova D, *et al*.,2020 |
| 99 | chr18:44085948 | c.5545G>A | p.Gly1849Arg | Exon36/40 | PLAT13 | Chinese  Czech | Uncertain Significance  (PM1+PM2+PP3+BP1) | Present study  Plevova P, *et al*.,2017 |
| 100 | chr18:44085885 | c.5608C>T | p.Arg1870Trp | Exon36/40 | PLAT13 | Japanese | Uncertain Significance  (PM1+PM2+PP3+BP1) | [Maekawa](https://pubmed.ncbi.nlm.nih.gov/?sort=date&size=20&term=Maekawa+K&cauthor_id=31547530) K, *et al*.,2019 |
| 101 | chr18:44085819 | c.5674G>T | p.Val1892Phe | Exon36/40 | PLAT13 | Japanese | Likely Pathogenic  (PM1+PM2+PP5+PP3+BP1) | Minami SB, *et al*.,2016 |
| 102 | chr18:44069064 | c.5734G>A | p.Asp1912Asn | Exon37/40 | PLAT13 | Japanese | Uncertain Significance  (PM1+PM2+PP3+BP1) | [Maekawa](https://pubmed.ncbi.nlm.nih.gov/?sort=date&size=20&term=Maekawa+K&cauthor_id=31547530) K, *et al*.,2019 |
| 103 | chr18:44068985 | c.5813G>A | p.Arg1938His | Exon37/40 | PLAT13 | Indian | Uncertain Significance  (PM1+PM2+PP3+BP1) | Chakrabarty S, *et al*.,2021 |
| 104 | chr18:44068983 | c.5815G>A | p.Asp1939Asn | Exon37/40 | PLAT13 | Chinese | Uncertain Significance  (PM1+PM2+PP3+BP1) | Hu S, *et al*., 2018 |
| 105 | chr18:44065109 | c.5869G>A | p.Glu1957Lys | Exon38/40 | PLAT14 | --- | Uncertain Significance  (PM1+PM2+PP3+BP1) | Sloan-Heggen CM, *et al*.,2016 |
| 106 | chr18:44065109 | c.5869G>T | p.Glu1957Ter | Exon38/40 | PLAT14 | Japanese | Pathogenic  (PVS1+PM2+PP3) | Mori K, *et al*.,2015 |
| 107 | chr18:44065093 | c.5885C>T | p.Thr1962Met | Exon38/40 | PLAT14 | Indian  Dutch | Likely Pathogenic  (PM1+PM2+PP5+PP3+BP1) | Wesdorp M, *et al*.,2018  Zazo Seco C, *et al*.,2017 |
| 108 | chr18:44065090 | c.5888delG | p.Gly1963AlafsTer136 | Exon38/40 | PLAT14 | Chinese | Pathogenic  (PVS1+PS1+PM2+PP3+PP5) | Present study  Bai X, *et al*.,2020 |
| 109 | chr18:44065084 | c.5894dup | p.Gly1966ArgfsTer28 | Exon38/40 | PLAT14 | Arab | Pathogenic  (PVS1+PM2+PP5) | Danial-Farran N, *et al*.,2018 |
| 110 | chr18:44065045 | c.5933G>A | p.Gly1978Asp | Exon38/40 | PLAT14 | Japanese | Uncertain Significance  (PM1+PM2+PP3+BP1) | [Maekawa](https://pubmed.ncbi.nlm.nih.gov/?sort=date&size=20&term=Maekawa+K&cauthor_id=31547530) K, *et al*.,2019 |
| 111 | chr18:44065044 | c.5934C>T | p.Gly1978= | Exon38/40 | PLAT14 | Dutch | Uncertain Significance  (PM2+BP4+BP7) | Wesdorp M, *et al*.,2018 |
| 112 | chr18:44065034 | c.5944C>T | p.Arg1982Ter | Exon38/40 | PLAT14 | --- | Pathogenic  (PVS1+PM2+PP5+PP3) | Sloan-Heggen CM, *et al*.,2016 |
| 113 | chr18:44065030 | c.5948C>T | p.Ser1983Phe | Exon38/40 | PLAT14 | Chinese | Uncertain Significance  (PM1+PM2+PP3+BP1) | [Shen](https://pubmed.ncbi.nlm.nih.gov/?sort=date&size=20&term=Shen+N&cauthor_id=30760222) N, *et al*.,2019 |
| 114 | chr18:44063668 | c.6037G>A | p.Gly2013Arg | Exon39/40 | PLAT14 | Japanese | Likely Pathogenic  (PM1+PM2+PM5+PP3+BP1) | [Maekawa](https://pubmed.ncbi.nlm.nih.gov/?sort=date&size=20&term=Maekawa+K&cauthor_id=31547530) K, *et al*.,2019 |
| 115 | chr18:44063667 | c.6038G>A | p.Gly2013Glu | Exon39/40 | PLAT14 | Chinese | Likely Pathogenic  (PM1+PM2+PP3+PP5+BP1) | Yu S, *et al*.,2021 |
| 116 | chr18:44063569 | c.6136G>A | p.Glu2046Lys | Exon39/40 | PLAT14 | Pakistani | Likely Pathogenic  (PM1+PM2+PP5+PP3+BP1) | Zhou Y, *et al*.,2020 |
| 117 | chr18:44057907 | c.3015_3017del  (NM_001145472.2)  [c.6162_6164del  (NM_144612.6)] | p.Phe1006del  p.Phe2055del | Exon40/40 | PLAT14 | --- | Likely Pathogenic  (PM1+PM2+PM4+PP3) | Sloan-Heggen CM, *et al*.,2016 |
| 118 | chr18:44057903 | c.6168del | p.Cys2057ValfsTer42 | Exon40/40 | PLAT14 | Japanese | Likely Pathogenic  (PVS1+PM2) | [Maekawa](https://pubmed.ncbi.nlm.nih.gov/?sort=date&size=20&term=Maekawa+K&cauthor_id=31547530) K, *et al*.,2019 |
| 119 | chr18:44057718 | c.6353G>A | p.Gly2118Glu | Exon40/40 | PLAT15 | Dutch  --- | Uncertain Significance  (PM2+PP3+BP1) | Wesdorp M, *et al*.,2018  Sloan-Heggen CM, *et al*.,2016 |
| 120 | chr18:44057716 | c.6355delG | p.Ala2119ProfsTer10 | Exon40/40 | PLAT15 | Chinese | Pathogenic  (PVS1+PM2+PP3) | Jin X, *et al*.,2022 |
| 121 | chr18:44057658 | c.6413G>A | p.Arg2138Gln | Exon40/40 | PLAT15 | Chinese | Uncertain Significance  (PM2+PP3+BP1) | Present study |
| 122 | chr18:44057599 | c.6472C>T | p.Arg2158Cys | Exon40/40 | PLAT15 | Czech | Uncertain Significance  (PM2+PP3+BP1) | Safka Brozkova D, *et al*.,2020 |
| 123 | chr18:44057557 | c.1417G>A  (NM_001145473.2)  [c.6514G>A  (NM_144612.6)] | p.Val473Met  [p.Val2172Met] | Exon9/9  [Exon40/40] | PLAT15 | Chinese | Uncertain Significance  (PM2+PP3+BP1) | Present study |
| 124 | chr18:44057475 | c.6598del | p.Asp2200MetfsTer22 | Exon40/40 | PLAT15 | --- | Likely Pathogenic  (PVS1+PM2+PP3) | Sloan-Heggen CM, *et al*.,2016 |
